# Highly Efficient Synthesis of Substituted 3,4-Dihydropyrimidin-2-(1*H*)-ones (DHPMs) Catalyzed by Hf(OTf)_4_: Mechanistic Insights into Reaction Pathways under Metal Lewis Acid Catalysis and Solvent-Free Conditions

**DOI:** 10.3390/molecules24020364

**Published:** 2019-01-21

**Authors:** Rui Kong, Shuai-Bo Han, Jing-Ying Wei, Xiao-Chong Peng, Zhen-Biao Xie, Shan-Shan Gong, Qi Sun

**Affiliations:** Jiangxi Key Laboratory of Organic Chemistry, Jiangxi Science and Technology Normal University, 605 Fenglin Avenue, Nanchang 330013, Jiangxi, China; kongrui0@126.com (R.K.); hanshuaibo1@163.com (S.-B.H.); weijingying1@126.com (J.-Y.W.); pengxiaochong6@126.com (X.-C.P.); xiezhenbiao1@126.com (Z.-B.X.)

**Keywords:** hafnium triflate, 3,4-dihydropyrimidin-2-(1*H*)-ones, solvent-free, Biginelli reaction, mechanism

## Abstract

In our studies on the catalytic activity of Group IVB transition metal Lewis acids, Hf(OTf)_4_ was identified as a highly potent catalyst for ”one-pot, three-component” Biginelli reaction. More importantly, it was found that solvent-free conditions, in contrast to solvent-based conditions, could dramatically promote the Hf(OTf)_4_-catalyzed formation of 3,4-dihydro-pyrimidin-2-(1*H*)-ones. To provide a mechanistic explanation, we closely examined the catalytic effects of Hf(OTf)_4_ on all three potential reaction pathways in both “sequential bimolecular condensations” and “one-pot, three-component” manners. The experimental results showed that the synergistic effects of solvent-free conditions and Hf(OTf)_4_ catalysis not only drastically accelerate Biginelli reaction by enhancing the imine route and activating the enamine route but also avoid the formation of Knoevenagel adduct, which may lead to an undesired byproduct. In addition, ^1^H-MMR tracing of the H-D exchange reaction of methyl acetoacetate in MeOH-*d*_4_ indicated that Hf(IV) cation may significantly accelerate ketone-enol tautomerization and activate the β-ketone moiety, thereby contributing to the overall reaction rate.

## 1. Introduction

As one of the most prominent multicomponent reactions (MCRs), Biginelli reaction has been utilized for the synthesis of 3,4-dihydropyrimidin-2-(1*H*)-ones (DHPMs) via acid-catalyzed one-pot condensation of aldehyde, β-ketoester, and urea in refluxing ethanol since its discovery in 1893 [1,2,3,4]. In past several decades, the identification of DHPM core structure in bioactive marine alkaloids including crambescidins [5], batzelladines [6], and monanchocidins [7], and a broad spectrum of pharmacological applications of both natural and synthetic DHPMs as antiviral, antibacterial, antitumor, anti-inflammatory, antihypertensive, and anti-epileptic agents, α_1A_ receptor antagonist, and calcium channel blocker, etc. [3,4,8] have elicited much attention to Biginelli reaction once again. In addition, DHPMs have also found applications as functional polymers [9], adhesives [10], and fabric dyes [11] in materials science.

Though the conventional Brønsted acid-catalyzed Biginelli reaction provides a reliable access to DHPMs, however, this method is limited by moderate yields and long reaction time. To improve the efficacy of Biginelli reaction, a large number of metal Lewis acids, such as FeCl_3_ [12], Yb(OTf)_3_ [13], Mn(OAc)_3_ [14], InCl_3_/InBr_3_/In(OTf)_3_ [15,16,17], ZrCl_4_/ZrOCl_2_·8H_2_O [18,19], CuCl_2_/Cu(OTf)_2_ [20,21], SmI_2_ [22], RuCl_3_ [23], Sr(OTf)_2_ [24], Ce(NO_3_)_3_ [25], TaBr_5_ [26], Bi(NO_3_)_2_ [27], SbCl_3_ [28], GaI_3_/Ga(OTf)_3_ [29,30], and TiCp_2_Cl_2_ [31] have been proved as effective catalysts for this purpose. Generally, the reaction conditions of metal Lewis acid-catalyzed DHPM synthesis fall into two categories. The first one is performed in organic solvents, such as EtOH, CH_3_CN, or tetrahydrofuran (THF). These methods typically need 10 mol% catalyst and 4–8 h to complete. The second one is conducted under solvent-free conditions, typically requires less catalyst (5%), and completes faster (0.5–1 h). In contrast, a Brønsted base-catalyzed Biginelli reaction (10 mol% *t*-BuOK) required much longer (3–8 h) to complete under the same solvent-free conditions [32]. 

In recent years, organometallic complexes of Bi(V) [33] and Cu(I) [34] have also been employed for the synthesis of DHPMs. In addition, the fast development of heterogeneous catalysts such as mesoporous materials [35], metal oxide nanoparticles [36], supported solid acids [37], and metal-coordinated polymers [38] has provided an alternative approach to DHPMs with improved catalyst recyclability. However, the preparation of these specific catalysts greatly limits their value in practical applications. In most of recent medicinal research, DHPMs were synthesized with easily accessible metal Lewis acid catalysts [39,40,41,42]. Therefore, the identification of highly potent, commercially available, affordable, and non-toxic metal Lewis acid catalysts for Biginelli reaction are still awaiting exploration.

Another fascinating aspect of Biginelli reaction is the revelation of reaction mechanism. The so-called imine, enamine, and Knoevenagel routes featuring different bimolecular condensation intermediates had been proposed [3,4]. In 1997, Kappe reexamined the mechanism and established that the imine route is the major pathway leading to the HCl-catalyzed formation of DHPMs [43], which are supported by the following mechanistic studies based on ESI-MS [44,45], NMR [45], and theoretical calculations [44,46]. However, most of the mechanisms proposed for metal Lewis acid-catalyzed Biginelli reactions were simply in favor of the imine route without much supporting evidence except Guo’s recent work on TiCp_2_Cl_2_ [31]. It should be noticed that Cepanec’s experimental results showed that SbCl_3_ inhibited both the imine and Knoevenagel routes and the reaction proceeded via the enamine route [28], which suggests that the mechanism of metal Lewis acid-catalyzed Biginelli reaction may vary from that based on HCl catalysis. Currently, more and more research has revealed that the reactions under solvent-free conditions could be of huge difference from those under solvent-based conditions in terms of yield, reaction rate, or even stereoselectivity [47,48]. However, the mechanistic explanation for the remarkably enhanced reactivity under solvent-free conditions over that in organic solvents has rarely been discussed in previous research.

Our ongoing research on the catalytic activity of Group IVB transition metal (Zr(IV) and Hf(IV)) salts showed that they have strong activation capability on carbonyl-transformation reactions, and Hf(IV) salts exhibit even superior reactivity to Zr(IV) salts in many reactions [49,50]. Inspired by these precedent results, we envisioned that Hf(IV) salts may promote Biginelli reaction via its strong interactions with aldehyde and β-ketoester. It has to be mentioned that, earlier, Chen et al. reported the catalytic activity of Hf(OTf)_4_ over several rare earth metal triflates on 2-selenoxo DHPM synthesis (5 mol%, 120 °C, 20 min–2 h) [51], whereas our independent research focused on the elucidation of reactivity of Hf(IV) salts on 2-oxo/thio DHPM synthesis and, more importantly, the underlying mechanism of catalysis. We report herein the identification of Hf(OTf)_4_ as a highly potent catalyst for Biginelli synthesis of 2-oxo/thio DHPMs under solvent-free conditions (1 mol%, 80 °C, 20–30 min). The catalytic effects of Hf(OTf)_4_ on all three potential reaction pathways were systematically investigated in both ‘sequential bimolecular condensations’ and ‘one-pot, three-component’ manners under solvent-free conditions. Our experimental results suggest that both the promotion of imine route and activation of enamine route by Hf(OTf)_4_ under solvent-free conditions are crucial for the marked enhancement of reaction efficacy.

## 2. Results and Discussion

### 2.1. Optimization of Hf(OTf)_4_-Catalyzed Biginelli Reaction Conditions

In the preliminary experiment, we compared the catalytic activity of Zr(IV) and Hf(IV) salts at 10 mol% level in a model reaction, which contained benzaldehyde, ethylacetoacetate, and urea in 1:1:1.2 molar ratio and was refluxed in ethanol. The results in Table 1 showed that ZrCl_4_- and ZrOCl_2_·8H_2_O-catalyzed reactions (~80%, 9–12 h) were significantly faster and higher-yielding than the conventional Biginelli reaction catalyzed by conc. HCl (65%, 18 h). ZrCp_2_Cl_2_ was less potent (62%, 18 h) than the other two Zr(IV) catalysts possibly due to the more hindered catalytic center. In contrast, HfCl_4_ (81%, 9 h) and HfCp_2_Cl_2_ (73%, 12 h) exhibited higher activity than their Zr(IV) counterparts, whereas Hf(OTf)_4_ appeared as the most potent catalyst and afforded **1** in 88% yield within only 9 h. 

In the following research, the solvent effect was investigated. As listed in Table 2, the reactions in toluene, dichloroethane, THF, and acetonitrile were hard to go to completion in the presence of 10 mol% Hf(OTf)_4_. In comparison, ethanol was a superior solvent for the Hf(OTf)_4_-catalyzed Biginelli reaction. To our surprise, when the reaction was performed under solvent-free conditions at 80 °C, it completed within only 5 min in 96% yield. Further lowering the amount of Hf(OTf)_4_ showed that as low as 1 mol% Hf(OTf)_4_ was sufficient enough to catalyze high-yielding conversion to **1** (95%, 20 min). We also noticed that the reaction could proceed at 80 °C in the absence of catalyst under solvent-free conditions, which is in agreement with a previous report [52]. However, the reaction was much slower and less efficient (63%, 8 h).

As another key factor for Biginelli reaction, the reaction temperature was varied to achieve the best result. It was found that lowering the temperature to 60 °C had negative effect on the reaction (87%, 40 min). When the temperature was increased to 100 °C, the reaction completed faster, but the yield dropped (76%, 5 min) due to the formation of polar byproducts. 

### 2.2. Scope of Hf(OTf)_4_-Catalyzed Biginelli Reaction under Solvent-Free Conditions 

With the optimized conditions, 1 mol% Hf(OTf)_4_ was applied to the synthesis of a diversity of 2-oxo/thio DHPMs. As shown in Table 3, benzaldehydes with electron-donating and electron-withdrawing groups (**2**–**5**), heteroarylaldehydes (**6**,**7**), and different β-ketoesters (**8**–**10**) were well tolerated by this method (>85%, 20−30 min). When acetylacetone (**11**), thiourea (**12**,**13**), or an aliphatic aldehyde (**14**) were employed, the corresponding DHPMs were obtained in only slightly lowered yields. 

### 2.3. Mechanistic Investigation on the Reaction Pathways of the Hf(OTf)_4_-Catalyzed Biginelli Reaction under Solvent-Free Conditions

Compared to that of metal Lewis acid-catalyzed Biginelli reactions performed in organic solvents, the mechanism of those under solvent-free conditions was simply interpreted as the same in most previous reports [13,20,24,29]. However, the huge difference in terms of catalyst needed, reaction rate, and yield between the two types of reaction conditions, as was observed in our and others’ research [13,23,24,25,27,30], urged us to reconsider how solvent-free conditions affect the outcome of Biginelli reaction from a mechanistic perspective. Our investigation started with the preparation of the three key intermediates **15**, **16**, and **17**, which correspond to the imine, enamine, and Knoevenagel routes, respectively. As described in previous reports, **15** is obtained as a white solid and poorly soluble in most organic solvent. Meanwhile, both **16** and **17** are found stable for TLC analysis. 

**Scheme 1:**
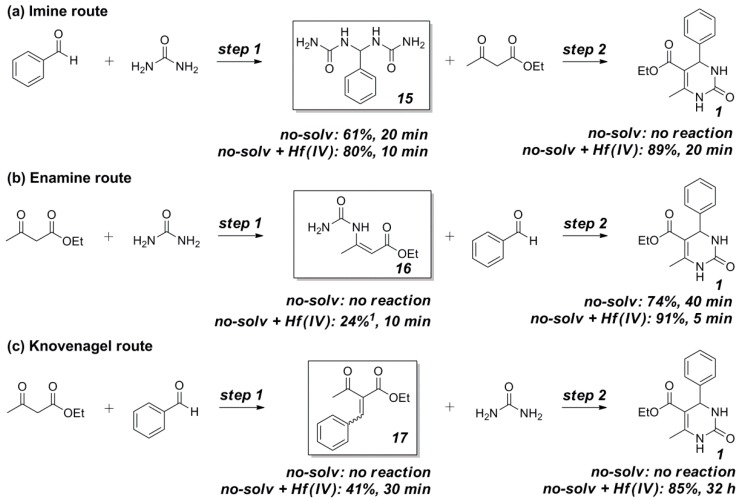
The three “sequential bimolecular condensation reactions” (**a**–**c**) under solvent-free conditions in the presence or absence of Hf(OTf)_4_. ^1^ Determined by ^1^H-NMR in DMSO-*d*_6_.

In the next stage, we closely analyzed the individual routes by tracing the sequential bimolecular condensations in the presence or absence of Hf(OTf)_4_ under solvent-free conditions. Compared to the reactions in ethanol (either with or without Hf(OTf)_4_), which barely generated much insoluble bisureide **15**, the bimolecular reaction between benzaldehyde and urea quickly formed a significant amount of white precipitation of **15** at 80 °C (61%, 20 min), indicating that the neat conditions promote the condensation (Scheme 1a). The presence of 1 mol% Hf(OTf)_4_ further enhanced the formation of **15** (80%, 10 min). However, in the second step, only in the presence of Hf(OTf)_4_ could **15** react with ethyl acetoacetate to form desired DHPM **1** (89%, 20 min). This result showed that Hf(OTf)_4_ catalyzed both the reversion of bisureide **15** back to the proposed imine intermediate **18** and its subsequent annulation with ethyl acetoacetate. The solvent-free conditions and the metal Lewis acid synergistically strengthen the imine pathway, which largely explains the remarkable acceleration of Hf(IV)-catalyzed Biginelli reaction under solvent-free conditions over that under solvent-based conditions. 

In solvent-based reaction, enamine **16** has been proved unable to react with benzaldehyde to yield **1** experimentally and theoretically. Therefore, the enamine route has not been regarded as a contribution in most previous research. However, we found that, under solvent-free conditions, Hf(OTf)_4_ could promote the quick condensation between ethyl acetoacetate and urea to give enamine **16** (24%, 10 min). The isolation by flash column chromatography indicated that **16** is easy to decompose in the presence of solvent, but is rather stable in solid form. More importantly, the subsequent annulation of **16** with benzaldehyde was fast and high-yielding in the presence of catalyst (91%, 5 min, Scheme 1b). This unexpected result strongly indicated that the presence of Hf(OTf)_4_ under solvent-free conditions activates the enamine route and opens the second pathway to reach the desired DHPM product. 

Then, the reaction between benzaldehyde and ethyl acetoacetate was examined. The data in Scheme 1c shows that Hf(OTf)_4_ plays a key role for the Knoevenagel condensation. Compared to that of **15** and **16**, the Hf(IV)-catalyzed formation of **17** under solvent-free conditions was slower (41%, 30 min). Moreover, its subsequent annulation with urea afforded **1** in an extremely slow rate (85%, 32 h). Considering that Hf(IV)-catalyzed ‘one-pot, three-component’ Biginelli reaction is quite fast (20 min) under solvent-free conditions, the Knoevenagel route could hardly be fitted into the reaction time frame of the ‘one-pot, three-component’ Biginelli reaction. 

**Figure 1:**
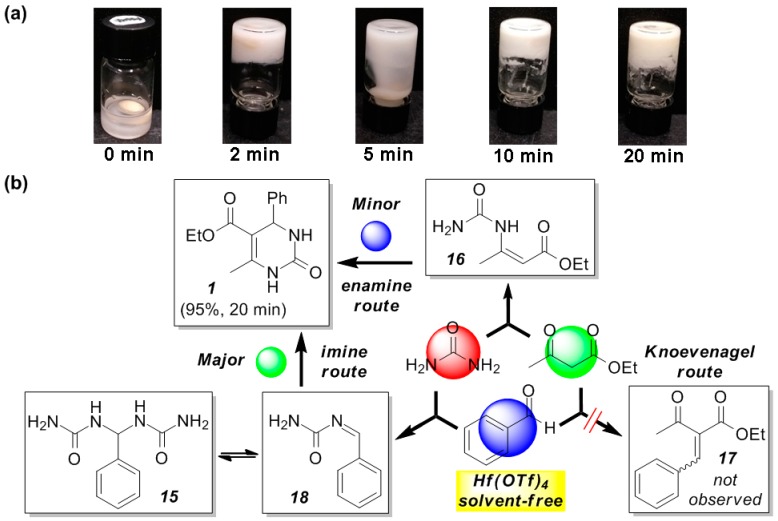
The photos (**a**) and proposed mechanism (**b**) for the Hf(OTf)_4_-catalyzed Biginelli reaction under solvent-free conditions.

Since the real “three-component” Biginelli reaction is more complicated, it was carefully reanalyzed under solvent-free conditions with Hf(OTf)_4_ to confirm the observations based on individual sequential bimolecular condensations. It was found that the originally clear reaction solution instantly generated a large amount of gel-like solid in the presence of Hf(IV) upon heating at 80 °C (2 min, Figure 1a). At this point, only a small amount of **1** and enamine **16** were observed on TLC, suggesting a large portion of the white solid obtained at this point might be bisureide, which was isolated and confirmed as **15**. As the reaction proceeded, the amount of white solid decreased and the viscosity of the reaction system dropped remarkably (5 min, Figure 1a) possibly due to the Hf(IV)-catalyzed reversion of **15** to imine intermediate **18**. Afterwards, as more and more DHPM **1** accumulated via the imine and enamine routes as white precipitate, the viscosity of the reaction system increased again (from 10 to 20 min, Figure 1a). Although it is hard to determine the contributions from imine and enamine routes precisely, however, the initial formation of a large amount of **15** strongly suggested that the imine route appears as the major pathway to the final DHPM. To our surprise, the Knoevenagel adduct **17** was not observed on TLC throughout the entire process, indicating that the Knoevenagel route is completely prohibited in competition with the other two routes (Figure 1b). 

In contrast, analysis of the “one-pot, three-component” Biginelli reaction without Hf(OTf)_4_ showed that Knoevenagel adduct **17** was observed due to the catalysis by urea as reported by Puripat and coworkers [48]. This result suggested that the imine and enamine routes are less competitive without Hf(OTf)_4_. However, based on the ‘sequential bimolecular condensations’ (Scheme 1C, step 2), **17** could not further react with urea to yield **1** without Hf(OTf)_4_. It was observed that as the amount of **17** decreased between 3 and 8 h, an unexpected byproduct **19**, the adduct of **17** and ethyl acetoacetate, began to accumulate and was isolated in 14% yield at the end. This result indicated that solvent-free conditions alone is not sufficient for the fast and high-yielding formation of **1**. The synergistic effect of both solvent-free conditions and Hf(OTf)_4_ catalysis not only drastically accelerated the Biginelli reaction via imine and enamine routes but also avoided the formation of Knoevenagel adduct, which may lead to an undesired byproduct.

**Figure 2:**
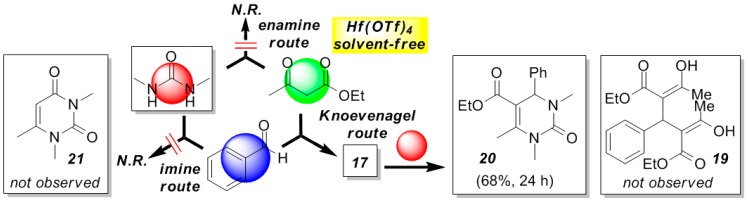
Mechanism variation for *N*,*N′*-dimethylurea-based Biginelli reaction.

Interestingly, when *N*,*N′*-dimethylurea, instead of urea, was applied as a substrate (Figure 2), the corresponding Hf(OTf)_4_-catalyzed reaction took much longer even under solvent-free conditions and afforded DHPM **20** in only moderate yield (68%, 24 h). Knoevenagel adduct **17** was the only intermediate observed on TLC. The simplified bimolecular condensation reactions showed that while *N*,*N′*-dimethylurea did not react with benzaldehyde at all, it could slowly react with ethyl acetoacetate to form a very small amount of pyrimidine **21** (10%, 24 h). However, in the three-component reaction, byproducts **19** and **21** were totally absent. These results showed that when both of the imine and enamine routes are blocked due to a certain specific substrate, the Knoevenagel route could dominate the reaction and eventually afford the DHPM product.

### 2.4. Activation Effect of Hf(OTf)_4_ on β-Ketoester 

**Figure 3:**
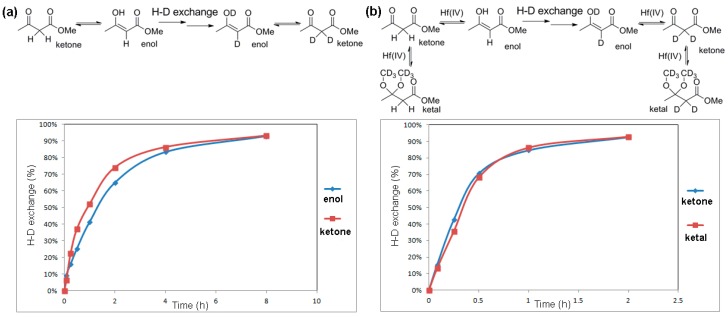
The H-D exchange reactions of methyl acetoacetate in MeOH-*d*_4_ in the absence (**a**) or presence (**b**) of Hf(OTf)_4_.

In our previous research, we have revealed Hf(IV) cation’s strong capability on activating benzaldehyde [50]. Many previous reports [13,14,20,24,29] had proposed that the interactions of metal cations with β-ketoester are also involved in the catalysis of Biginelli reaction. However, not much evidence has been provided to support this point. In current research, we utilized ^1^H-NMR to examine the activation effects of Hf(IV) on β-ketoester (Supplementary Materials, Figure S43–S44). Interestingly, when 5 mol% of Hf(OTf)_4_ was added to methyl acetoacetate in MeOH-*d*_4_, the rate of H-D exchange was remarkably promoted. As shown in Figure 3b, 93% of the proton at active methylene position was exchanged to deuterium in only 2 h. In the control experiment, it took 8 h for the H-D exchange to reach the equilibrium at 93% without Hf(OTf)_4_ (Figure 3a). Similar to the promoted tautomerization of dimethylphosphite in Kabachnik reaction [50], Hf(IV) accelerated the tautomerization of methylacetoacetate between the ketone and enol forms, thereby increasing overall reaction rate. More importantly, it was observed that Hf(IV) also activated the β-ketone moiety and induced the formation of ketal, which accounted for ~55% of the total β-ketoester used. This result is in good agreement with our observations that all steps involving benzaldehyde and ethyl acetoacetate (Scheme 1) are largely promoted. 

## 3. Materials and Methods 

### 3.1. General Methods

General chemical reagents and solvents were obtained from commercial suppliers. All reactions were monitored by thin layer chromatography on plates coated with 0.25 mm silica gel 60 F254 (Qingdao Haiyang Chemicals, Qingdao, China). TLC plates were visualized by UV irradiation (254 nm, Shanghai Peiqing Sci & Tech, Shanghai, China). Flash column chromatography employed silica gel (particle size 32–63 μm, Qingdao Haiyang Chemicals, Qingdao, China). Melting points were determined with a Thomas-Hoover melting point apparatus and uncorrected (Thomas Scientific, Swedesboro, NJ, USA). NMR spectra were obtained with a Bruker AV-400 instrument (Bruker BioSpin, Faellanden, Switzerland) with chemical shifts reported in parts per million (ppm, *δ*) and referenced to CDCl_3_ or DMSO-*d*_6_. The NMR spectra of compounds **1**–**17** and **19**–**21** were provided in Supplementary Materials (Figures S1–S42). IR spectra were recorded on a Bruker Vertex-70 spectrometer (Bruker Optics, Billerica, MA, USA). Low-resolution mass spectra were reported as *m/z* and obtained with a Bruker amaZon SL mass spectrometer (Bruker Daltonics, Billerica, MA, USA). 

### 3.2. General Synthetic Procedure and Characterization of DHPMs ***1***–***14*** and ***20***

To a mixture of aldehyde (1.0 mmol), β-ketoester/diketone (1.0 mmol), and urea/thiourea (1.2 mmol) was added Hf(OTf)_4_ (0.01 mmol). The reaction was stirred at 80 °C for 20–30 min. Then ethanol (2 mL) was added to the reaction to dissolve the solid residue completely. Upon cooling, the solid precipitate was filtered, washed, and dried to afford pure DHPM product. For compounds **9**, **10**, **13**, **14**, and **20**, the crude product was directly dissolved in CH_2_Cl_2_ and purified by flash column chromatography.

*5-Ethoxycarbonyl-6-methyl-4-phenyl-3,4-dihydropyrimidin-2(1H)-one* (**1**). The reaction of benzaldehyde (106 mg, 1 mmol), ethyl acetoacetate (130 mg, 1 mmol), urea (72 mg, 1.2 mmol), and Hf(OTf)_4_ (8 mg, 0.01 mmol) afforded **1** (247 mg, 95%) as a white solid; mp 209−210 °C. ^1^H-NMR(400 MHz, DMSO-*d*_6_): *δ* 9.18 (s, 1H), 7.73 (s, 1H), 7.32−7.25 (m, 5H), 5.14 (s, 1H), 3.98 (q, *J* = 6.8 Hz, 2H), 2.25 (s, 3H), 1.09 (t, *J* = 6.8 Hz, 3H) ppm; ^13^C-NMR (100 MHz, DMSO-*d*_6_): *δ* 165.3, 152.1, 148.3, 144.8, 128.3 (×2), 127.2, 126.2 (×2), 99.2, 59.1, 53.9, 17.7, 14.0 ppm; IR (KBr): *ν*_max_ 3248, 3121, 1725, 1699, 1648, 1420, 1314, 1221, 1090, 698 cm^−^^1^; LRMS (ESI+): *m/z* calcd for C_14_H_16_N_2_O_3_ [M + H]^+^ 261.1; found 261.2.

*5-Ethoxycarbonyl-6-methyl-4-(4-methylphenyl)-3,4-dihydropyrimidin-2(1H)-one* (**2**). The reaction of 4-methylbenzaldehyde (120 mg, 1 mmol), ethyl acetoacetate (130 mg, 1 mmol), urea (72 mg, 1.2 mmol), and Hf(OTf)_4_ (8 mg, 0.01 mmol) afforded **2** (252 mg, 92%) as a white solid; mp 218−219 °C. ^1^H-NMR(400 MHz, DMSO-*d*_6_): *δ* 9.15 (s, 1H), 7.67 (s, 1H), 7.15 (d, *J* = 8.4 Hz, 2H), 6.87 (d, *J* = 8.4 Hz, 2H), 5.10 (d, *J* = 2.6 Hz, 1H), 3.98 (q, *J* = 7.1 Hz, 2H), 3.71 (s, 3H), 2.24 (s, 3H), 1.10 (t, *J* = 7.1 Hz, 3H) ppm; ^13^C-NMR (100 MHz, DMSO-*d*_6_): *δ* 165.4, 158.4, 152.1, 147.9, 137.0, 127.4 (×2), 113.7 (×2), 99.6, 59.1, 55.0, 53.3, 17.7, 14.1 ppm; IR (KBr): *ν*_max_ 3244, 3113, 2937, 1723, 1704, 1650, 1460, 1309, 1286, 1222, 1088, 783 cm^−1^; LRMS (ESI+): *m/z* calcd for C_15_H_18_N_2_O_3_ [M + H]^+^ 275.1; found 275.2.

*5-Ethoxycarbonyl-4-(4-methoxyphenyl)-6-methyl-3,4-dihydropyrimidin-2(1H)-one* (**3**). The reaction of 4-methoxybenzaldehyde (136 mg, 1 mmol), ethyl acetoacetate (130 mg, 1 mmol), urea (72 mg, 1.2 mmol), and Hf(OTf)_4_ (8 mg, 0.01 mmol) afforded **3** (270 mg, 93%) as a white solid; mp 201−202 °C. ^1^H-NMR(400 MHz, DMSO-*d*_6_): *δ* 9.17 (s, 1H), 7.70 (s, 1H), 7.12 (s, 4H), 5.12 (s, 1H), 3.98 (q, *J* = 7.0 Hz, 2H), 2.25 (s, 6H), 1.10 (t, *J* = 7.0 Hz, 3H) ppm; ^13^C-NMR (100 MHz, DMSO-*d*_6_): *δ* 165.4, 158.4, 152.1, 147.9, 137.0, 127.4 (×2), 113.7 (×2), 99.6, 59.1, 55.0, 53.3, 17.7, 14.1 ppm; IR (KBr): *ν*_max_ 3250, 3113, 2940, 1714, 1695, 1652, 1440, 1306, 1279, 1220, 1085, 769 cm^−1^; LRMS (ESI+): *m/z* calcd for C_15_H_18_N_2_O_4_ [M + H]^+^ 291.1; found 291.0.

*4-(4-Chlorophenyl)-5-ethoxycarbonyl-6-methyl-3,4-dihydropyrimidin-2(1H)-one* (**4**). The reaction of 4-chlorobenzaldehyde (140 mg, 1 mmol), ethyl acetoacetate (130 mg, 1 mmol), urea (72 mg, 1.2 mmol), and Hf(OTf)_4_ (8 mg, 0.01 mmol) afforded **4** (259 mg, 88%) as a yellow solid; mp 215−216 °C. ^1^H-NMR(400 MHz, DMSO-*d*_6_): *δ* 9.26 (s, 1H), 7.78 (s, 1H), 7.39 (d, *J* = 8.1 Hz, 2H), 7.26 (d, *J* = 8.1 Hz, 2H), 5.16 (s, 1H), 3.98 (q, *J* = 7.0 Hz, 2H), 2.26 (s, 3H), 1.09 (t, *J* = 7.0 Hz, 3H) ppm; ^13^C-NMR (100 MHz, DMSO-*d*_6_): *δ* 165.2, 151.9, 148.6, 143.7, 131.8, 128.3 (×2), 128.1 (×2), 98.9, 59.2, 53.4, 17.7, 14.0 ppm; IR (KBr): *ν*_max_ 3248, 3113, 2933, 1725, 1707, 1649, 1313, 1290, 1222, 1091, 698 cm^−1^; LRMS (ESI+): *m/z* calcd for C_14_H_15_ClN_2_O_3_ [M + H]^+^ 295.1; found 295.1.

*5-Ethoxycarbonyl-6-methyl-4-(4-nitrophenyl)-3,4-dihydropyrimidin-2(1H)-one* (**5**). The reaction of 4-nitrobenzaldehyde (151 mg, 1 mmol), ethyl acetoacetate (130 mg, 1 mmol), urea (72 mg, 1.2 mmol), and Hf(OTf)_4_ (8 mg, 0.01 mmol) afforded **5** (259 mg, 85%) as a yellow solid; mp 212−213 °C. ^1^H-NMR(400 MHz, DMSO-*d*_6_): *δ* 9.36 (s, 1H), 8.21 (d, *J* = 8.4 Hz, 2H), 7.90 (s, 1H) 7.51 (d, *J* = 8.4 Hz, 2H), 5.29 (s, 1H), 3.98 (q, *J* = 7.0 Hz, 2H), 2.27 (s, 3H), 1.09 (t, *J* = 7.0 Hz, 3H) ppm; ^13^C-NMR (100 MHz, DMSO-*d*_6_): *δ* 165.0, 152.0, 151.7, 149.3, 146.7, 127.6 (×2), 123.7 (×2), 98.2, 59.3, 53.7, 17.8, 14.0 ppm; IR (KBr): *ν*_max_ 3240, 3116, 2964, 1729, 1707, 1646, 1520, 1348, 1216, 1008, 782, 697 cm^−1^; LRMS (ESI+): *m/z* calcd for C_14_H_15_N_3_O_5_[M + H]^+^ 306.1; found 306.1.

*5-Ethoxycarbonyl-4-(2-furanyl)-6-methyl-3,4-dihydropyrimidin-2(1H)-one* (**6**). The reaction of furfural (96 mg, 1 mmol), ethyl acetoacetate (130 mg, 1 mmol), urea (72 mg, 1.2 mmol), and Hf(OTf)_4_ (8 mg, 0.01 mmol) afforded **6** (228 mg, 91%) as a white solid; mp 209−210 °C. ^1^H-NMR(400 MHz, DMSO-*d*_6_): *δ* 9.25 (s, 1H), 7.76 (s, 1H), 7.55 (s, 1H), 6.36−6.35 (m, 1H), 6.09 (d, *J* = 3.0 Hz, 1H), 5.20 (d, *J* = 3.3 Hz, 1H), 4.05−4.00 (m, 2H), 2.23 (s, 3H), 1.13 (t, *J* = 7.1 Hz, 3H) ppm; ^13^C-NMR (100 MHz, DMSO-*d*_6_): *δ* 165.0, 156.0, 152.4, 149.3, 142.1, 110.3, 105.2, 96.8, 59.2, 47.7, 17.7, 14.1 ppm; IR (KBr): *ν*_max_ 3244, 3102, 1701, 1649, 1318, 1234, 1100, 740 cm^−1^; LRMS (ESI+): *m/z* calcd for C_12_H_14_N_2_O_4_ [M + H]^+^ 251.1; found 251.1.

*5-Ethoxycarbonyl-4-(2-thienyl)-6-methyl-3,4-dihydropyrimidin-2(1H)-one* (**7**). The reaction of 2-thenaldehyde (112 mg, 1 mmol), ethyl acetoacetate (130 mg, 1 mmol), urea (72 mg, 1.2 mmol), and Hf(OTf)_4_ (8 mg, 0.01 mmol) afforded **7** (253 mg, 95%) as a white solid; mp 216−217 °C. ^1^H-NMR(400 MHz, DMSO-*d*_6_): *δ* 9.33 (s, 1H), 7.92 (s, 1H), 7.34 (d, *J* = 5.0 Hz, 1H), 6.95−6.90 (m, 2H), 5.42 (d, *J* = 3.3 Hz, 1H), 4.06 (q, *J* = 7.0 Hz, 2H), 2.22 (s, 3H), 1.16 (t, *J* = 7.0 Hz, 3H) ppm; ^13^C-NMR (100 MHz, DMSO-*d*_6_): *δ* 165.0, 152.2, 148.8, 148.6, 126.6, 124.5, 123.4, 99.8, 59.3, 49.3, 17.6, 14.1 ppm; IR (KBr): *ν*_max_ 3334, 3237, 3120, 1703, 1650, 1317, 1225, 1096, 789 cm^−1^; LRMS (ESI+): *m/z* calcd for C_12_H_14_N_2_O_3_S [M + H]^+^ 267.1; found 267.0.

*5-Methoxycarbonyl-6-methyl-4-phenyl-3,4-dihydropyrimidin-2(1H)-one* (**8**). The reaction of benzaldehyde (106 mg, 1 mmol), methyl acetoacetate (116 mg, 1 mmol), urea (72 mg, 1.2 mmol), and Hf(OTf)_4_ (8 mg, 0.01 mmol) afforded **8** (236 mg, 96%) as a white solid; mp 216−217 °C. ^1^H-NMR(400 MHz, DMSO-*d*_6_): *δ* 9.25 (s, 1H), 7.78 (s, 1H), 7.34–7.23 (m, 5H), 5.15 (d, *J* = 3.3 Hz, 1H), 3.53 (s, 3H), 2.26 (s, 3H) ppm; ^13^C-NMR (100 MHz, DMSO-*d*_6_): *δ* 165.8, 152.2, 148.6, 144.6, 128.4 (×2), 127.2, 126.1 (×2), 99.0, 53.8, 50.7, 17.8 ppm; IR (KBr): *ν*_max_ 3338, 2950, 1713, 1680, 1661, 1642, 1431, 1380, 1346, 1280, 1237, 1088, 755, 700 cm^−1^; LRMS (ESI+): *m/z* calcd for C_13_H_14_N_2_O_3_ [M + H]^+^ 247.1; found 247.1.

*5-Ethoxycarbonyl-4-(4-methylphenyl)-6-propyl-3,4-dihydropyrimidin-2(1H)-one* (**9**). The reaction of 4-methylbenzaldehyde (120 mg, 1 mmol), ethyl butyrylacetate (158 mg, 1 mmol), urea (72 mg, 1.2 mmol), and Hf(OTf)_4_ (8 mg, 0.01 mmol) afforded (PE/EA = 5:4) **9** (269 mg, 89%) as a glassy solid; mp 146−147 °C. ^1^H-NMR(400 MHz, DMSO-*d*_6_): *δ* 9.13 (s, 1H), 7.67 (s, 1H), 7.11 (s, 4H), 5.10 (d, *J* = 2.6 Hz, 1H), 3.97 (q, *J* = 7.0 Hz, 2H), 2.62 (t, *J* = 7.3 Hz, 2H), 2.25 (s, 3H), 1.55 (q, *J* = 7.3 Hz, 2H), 1.10 (t, *J* = 7.0 Hz, 3H), 0.90 (t, *J* = 7.3 Hz, 3H) ppm; ^13^C-NMR (100 MHz, DMSO-*d*_6_): *δ* 165.1, 152.3, 152.1, 142.0, 136.3, 128.8 (×2), 126.1 (×2), 99.2, 59.1, 53.6, 32.3, 21.6, 20.6, 14.0, 13.6 ppm; IR (KBr): *ν*_max_ 3252, 3121, 1702, 1643, 1308, 1210, 1084, 796 cm^−1^; LRMS (ESI+): *m/z* calcd for C_17_H_22_N_2_O_3_ [M + H]^+^ 303.2; found 303.2.

*5-Ethoxycarbonyl-6-ethyl-4-(4-methoxyphenyl)-3,4-dihydropyrimidin-2(1H)-one* (**10**). The reaction of 4-methoxybenzaldehyde (136 mg, 1 mmol), ethyl propionylacetate (144 mg, 1 mmol), urea (72 mg, 1.2 mmol), and Hf(OTf)_4_ (8 mg, 0.01 mmol) afforded (PE/EA = 5:4) **10** (252 mg, 83%) as a glassy solid; mp 151−152 °C. ^1^H-NMR(400 MHz, CDCl_3_): *δ* 8.61 (s, 1H), 7.22−6.80 (m, 4H), 6.25 (s, 1H), 5.31 (s, 1H), 4.05 (q, *J* = 6.9 Hz, 2H), 3.76 (s, 3H), 2.74−2.64 (m, 2H), 1.25−1.14 (m, 6H) ppm; ^13^C-NMR (100 MHz, CDCl_3_): *δ* 165.4, 159.1, 154.2, 151.8, 136.3, 127.7 (×2), 113.9 (×2), 100.6, 59.9, 55.2, 54.8, 25.2, 14.1, 12.6 ppm; IR (KBr): *ν*_max_ 3386, 3241, 3103, 2978, 1702, 1646, 1511, 1460, 1302, 1220, 1173, 797 cm^−1^; LRMS (ESI+): *m/z* calcd for C_16_H_20_N_2_O_4_ [M + H]^+^ 305.1; found 305.2.

*5-Acetyl-6-methyl-4-phenyl-3,4-dihydropyrimidin-2(1H)-one* (**11**). The reaction of benzaldehyde (106 mg, 1 mmol), 2,4-pentanedione (100 mg, 1 mmol), urea (72 mg, 1.2 mmol), and Hf(OTf)_4_ (8 mg, 0.01 mmol) afforded **11** (253 mg, 95%) as a white solid; mp 239−240 °C. ^1^H-NMR(400 MHz, DMSO-*d*_6_): *δ* 9.21 (s, 1H), 7.85 (s, 1H), 7.34–7.24 (m, 5H), 5.26 (d, *J* = 2.6 Hz, 1H), 2.29 (s, 3H), 2.10 (s, 3H) ppm; ^13^C-NMR (100 MHz, DMSO-*d*_6_): *δ* 194.2, 152.1, 148.0, 144.2, 128.5 (×2), 127.3, 126.4 (×2), 109.6, 53.8, 30.2, 18.8 ppm; IR (KBr): *ν*_max_ 3258, 1712, 1702, 1675, 1455, 1366, 1330, 1236, 769 cm^−1^; LRMS (ESI+): *m/z* calcd for C_13_H_14_N_2_O_2_ [M + H]^+^ 231.1; found 231.1.

*5-Ethoxycarbonyl-6-methyl-4-phenyl-3,4-dihydropyrimidin-2(1H)-thione* (**12**). The reaction of benzaldehyde (106 mg, 1 mmol), ethyl acetoacetate (130 mg, 1 mmol), thiourea (91 mg, 1.2 mmol), and Hf(OTf)_4_ (8 mg, 0.01 mmol) afforded **12** (226 mg, 82%) as a yellow solid; mp 208−209 °C. ^1^H-NMR(400 MHz, DMSO-*d*_6_): *δ* 10.34 (s, 1H), 9.66 (s, 1H), 7.36-7.21 (m, 5H), 5.18 (d, *J* = 2.6 Hz, 1H), 4.00 (q, *J* = 7.0 Hz, 2H), 2.29 (s, 3H), 1.10 (t, *J* = 7.0 Hz, 3H) ppm; ^13^C-NMR (100 MHz, DMSO-*d*_6_): *δ* 174.3, 165.1, 145.0, 143.5, 128.5 (×2), 127.7, 126.4 (×2), 100.7, 59.6, 54.0, 17.1, 14.0 ppm; IR (KBr): *ν*_max_ 3327, 3172, 3099, 1982, 1669, 1574, 1465, 1327, 1283, 1176, 1118, 760, 693 cm^−1^; LRMS (ESI+): *m/z* calcd for C_14_H_1__6_N_2_O_2_S [M + H]^+^ 277.1; found 277.1.

*5-Ethoxycarbonyl-6-ethyl-4-phenyl-3,4-dihydropyrimidin-2(1H)-thione* (**13**). The reaction of benzaldehyde (106 mg, 1 mmol), ethyl propionylacetate (144 mg, 1 mmol), thiourea (91 mg, 1.2 mmol), and Hf(OTf)_4_ (8 mg, 0.01 mmol) afforded (PE/EA = 5:3) **13** (232 mg, 80%) as a glassy solid; mp 156−157 °C. ^1^H-NMR(400 MHz, CDCl_3_): *δ* 8.57 (s, 1H), 7.97 (s, 1H), 7.31−7.24 (m, 5H), 5.35 (d, *J* = 3.1 Hz, 1H), 4.09−4.01 (m, 2H), 2.73 (q, *J* = 7.5 Hz, 2H), 1.21−1.11 (m, 6H) ppm; ^13^C-NMR (100 MHz, CDCl_3_): *δ* 174.6, 164.9, 148.5, 142.5, 128.9, 128.5, 128.3, 127.0, 126.8, 102.0, 60.4, 56.0, 24.8, 14.0, 12.0 ppm; IR (KBr): *ν*_max_ 3179, 2982, 1705, 1649, 1474, 1185, 1099, 762, 696 cm^−1^; LRMS (ESI+): *m/z* calcd for C_15_H_1__8_N_2_O_2_S [M + H]^+^ 291.1; found 291.1.

*5-Ethoxycarbonyl-6-methyl-4-pentyl-3,4-dihydropyrimidin-2(1H)-one* (**14**). The reaction of *n*-hexaldehyde (100 mg, 1 mmol), ethyl acetoacetate (130 mg, 1 mmol), urea (72 mg, 1.2 mmol), and Hf(OTf)_4_ (8 mg, 0.01 mmol) afforded (PE/EA = 2:1) **14** (209 mg, 82%) as a white solid; mp 151−152 °C. ^1^H-NMR(400 MHz, CDCl_3_): *δ* 8.21 (s, 1H), 5.85 (s, 1H), 4.30−4.13 (m, 3H), 2.28 (s, 3H), 1.52 (m, 2H), 1.42−1.25 (m, 9H), 0.86 (t, *J* = 6.6 Hz, 3H) ppm; ^13^C-NMR (100 MHz, CDCl_3_): *δ* 166.0, 154.6, 146.7, 101.8, 60.0, 51.7, 36.9, 31.5, 24.1, 22.6, 18.6, 14.4, 14.1 ppm; IR (KBr): *ν*_max_ 3245, 3121, 2954, 1725, 1420, 1314, 1221, 1090, 698 cm^−1^; LRMS (ESI+): *m/z* calcd for C_1__3_H_22_N_2_O_3_ [M +H ]^+^ 255.3; found 255.3.

*5-Ethoxycarbonyl-1,3,6-trimethyl-4-phenyl-3,4-dihydropyrimidin-2(1H)-one* (**20**). The reaction of benzaldehyde (106 mg, 1 mmol), ethyl acetoacetate (130 mg, 1 mmol), *N,N’*-dimethyl urea (106 mg, 1.2 mmol), and Hf(OTf)_4_ (8 mg, 0.01 mmol) afforded **20** (199 mg, 68%) as colorless oil. ^1^H-NMR(400 MHz, CDCl_3_): *δ* 7.30−7.21 (m, 5H), 5.24 (s, 1H), 4.16−4.11 (m, 2H), 3.26 (s, 3H), 2.91 (s, 3H), 2.48 (s, 3H), 1.24 (t, *J* = 7.1 Hz, 3H) ppm; ^13^C-NMR (100 MHz, CDCl_3_): *δ* 166.0, 153.9, 149.2, 141.1, 128.6 (×2), 127.9, 126.6 (×2), 103.7, 60.9, 60.1, 34.4, 31.0, 16.6, 14.2 ppm; IR (KBr): *ν*_max_ 3240, 3110, 2980, 1710, 1665, 1316, 1220, 1097, 780 cm^−1^; LRMS (ESI+): *m/z* calcd for C_16_H_20_N_2_O_3_ [M + H]^+^ 289.1; found 289.1. 

### 3.3. Synthetic Procedures and Characterization of ***15***–***17***, ***19***, and ***21***

*1,1’-Phenylmethanediyl-bis-urea* (**15**). To the mixture of benzaldehyde (106 mg, 1 mmol) and urea (72 mg, 1.2 mmol) was added Hf(OTf)_4_ (8 mg, 0.01 mmol). The reaction was stirred at 80 °C for 10 min. Upon cooling, the white solid residue was washed with CH_2_Cl_2_ (×3) and EtOH (×3) to afford **15** (111 mg, 80%) as a white solid; mp 216−218 °C. ^1^H-NMR(400 MHz, DMSO-*d*_6_): *δ* 7.33 (s, 5H), 7.26 (s, 1H), 6.94 (ds, 1H), 6.77 (ds, 1H), 6.18 (ds, 2H), 5.66 (s, 2H) ppm; ^13^C-NMR (100 MHz, DMSO-*d*_6_): *δ* 157.6, 157.5, 142.5, 128.0 (×2), 127.0, 125.9 (×2), 59.1 ppm; LRMS (ESI+): *m/z* calcd for C_9_H_12_N_4_O_2_ [M + H]^+^ 209.1; found 209.1.

*3-Ureido-crotonic acid ethyl ester* (**16**). To the mixture of ethyl acetoacetate (130 mg, 1 mmol) and urea (72 mg, 1.2 mmol) was added Hf(OTf)_4_ (8 mg, 0.01 mmol). The reaction was stirred at 80 °C for 10 min. (The yield of **16** in the reaction mixture was determined to be 24% by ^1^H-NMR in DMSO-*d*_6_ when reaction reached equilibrium.) Flash column chromatography afforded (PE/EA = 4:1) **16** (9 mg, 5%) as a white solid (due to partial decomposition during purification); mp 165−166 °C. ^1^H-NMR(400 MHz, CDCl_3_): *δ* 10.15 (s, 1H), 6.80 (bs, 2H), 4.76 (s, 1H), 4.06 (q, *J* = 7.0 Hz, 2H), 2.24 (s, 3H), 1.18 (t, *J* = 7.1 Hz, 3H) ppm; ^13^C-NMR (100 MHz, CDCl_3_): *δ* 168.2, 156.7, 154.1, 91.7, 58.7, 21.5, 14.2 ppm; LRMS (ESI+): *m/z* calcd for C_7_H_12_N_2_O_3_ [M + H]^+^ 173.1; found 173.1.

*(E)-2-Acetyl-3-phenylacrylic acid ethyl ester* (**17a**). To the mixture of benzaldehyde (106 mg, 1 mmol) and ethyl acetoacetate (130 mg, 1 mmol) was added Hf(OTf)_4_ (8 mg, 0.01 mmol). The reaction was stirred at 80 °C for 30 min. Flash column chromatography afforded (PE/DCM = 5:4) **17a** (89 mg, 20%) as yellow oil. ^1^H-NMR(400 MHz, CDCl_3_): *δ* 7.66 (s, 1H), 7.37 (s, 5H), 4.29 (q, *J* = 7.1 Hz, 2H), 2.34 (s, 3H), 1.32 (t, *J* = 7.1 Hz, 3H) ppm; ^13^C-NMR (100 MHz, CDCl_3_): *δ* 203.2, 164.5, 140.5, 134.3, 133.0, 130.4, 129.7 (×2), 129.0 (×2), 61.6, 31.2, 14.2 ppm; LRMS (ESI+): *m/z* calcd for C_13_H_1__4_O_3_ [M + H]^+^ 219.1; found 219.2.

*(Z)-2-Acetyl-3-phenylacrylic acid ethyl ester* (**17b**). After **17a** was eluted from the column, more polar eluent afforded (PE/EA = 10:1) **17b** (89 mg, 21%) as yellow oil. ^1^H-NMR(400 MHz, CDCl_3_): *δ* 7.57 (s, 1H), 7.46−7.37 (m, 5H), 4.33 (q, *J* = 7.2 Hz, 2H), 2.42 (s, 3H ), 1.27 (t, *J* = 7.2 Hz, 3H) ppm; ^13^C-NMR (100 MHz, CDCl_3_): *δ* 194.6, 167.7, 141.2, 134.7, 133.0, 130.7, 129.5 (×2), 128.8 (×2), 61.7, 26.5, 13.8 ppm; LRMS (ESI+): *m/z* calcd for C_13_H_14_O_3_ [M + H]^+^ 219.1; found 219.2. 

*(2Z,4Z)-Diethyl 2,4-bis(1-hydroxyethylidene)-3-phenylpentanedioate* (**19**). The mixture of benzaldehyde (106 mg, 1 mmol), ethyl acetoacetate (130 mg, 1 mmol), and urea (72 mg, 1.2 mmol) was stirred at 80 °C for 24 h. Flash column chromatography afforded (PE/EA = 5:2) **19** (24 mg, 14%) as a white solid; mp 160−161 °C. ^1^H-NMR(400 MHz, CDCl_3_): *δ* 7.29−7.10 (m, 5H), 5.89 (s, 1H), 4.89 (s, 1H), 4.12−4.04 (m, 4H), 2.31 (s, 6H ), 1.22 (t, *J* = 7.1 Hz, 6H) ppm; ^13^C-NMR (100 MHz, CDCl_3_): *δ* 167.7 (×2), 147.8, 144.0 (×2), 128.0 (×2), 127.9 (×2), 126.1, 104.2 (×2), 59.8 (×2), 39.7, 19.5 (×2), 14.3 (×2) ppm; LRMS (ESI+): *m/z* calcd for C_19_H_24_O_6_ [M + H]^+^ 349.2; found 349.2. 

*1,3,6-Trimethyluracil* (**21**). To the mixture of ethyl acetoacetate (130 mg, 1 mmol) and urea (72 mg, 1.2 mmol) was added Hf(OTf)_4_ (8 mg, 0.01 mmol). The reaction was stirred at 80 °C for 24 h. Flash column chromatography afforded (DCM/EA = 5:2) **21** (15 mg, 10%) as a white solid; mp 115−116 °C. ^1^H-NMR(400 MHz, CDCl_3_): *δ* 5.16 (s, 1H), 3.39 (s, 3H), 3.32 (s, 3H), 2.22 (s, 3H) ppm; ^13^C-NMR (100 MHz, CDCl_3_): *δ* 162.4, 152.6, 151.4, 101.2, 31.7, 27.9, 20.2 ppm; LRMS (ESI+): *m/z* calcd for C_7_H_10_N_2_O_2_ [M + H]^+^ 155.1; found 155.1. 

## 4. Conclusions

In summary, Hf(OTf)_4_ was identified as a highly efficient catalyst for Biginelli reaction. Under solvent-free conditions, as low as 1 mol% Hf(OTf)_4_ could catalyze efficient formation of a diversity of 2-oxo/thio DHPMs in only 20–30 min. The mechanistic investigation based on ‘sequential bimolecular condensations’ showed that the enhancement of imine route and unexpected activation of enamine route are responsible for the dramatically improved catalytic activity of Hf(OTf)_4_ under solvent-free conditions over that under solvent-based conditions. The tracing of ‘one-pot, three-component’ Biginelli reaction not only confirmed our observations on individual routes but also revealed the formation of bisureide **15** as a transient intermediate and total inhibition of Knoevenagel route under Hf(OTf)_4_ catalysis and solvent-free conditions. However, in the case of an *N*,*N′*-dimethylurea-based Biginelli reaction, the corresponding DHPM **20** was obtained almost solely via Knoevenagel route rather than the prohibited imine and enamine routes in a much slower rate. These results strongly suggest that the mechanism for Biginelli reaction may vary depending on both reaction conditions and reactivity of substrates, and should not be simply interpreted as the same as the classic Biginelli reaction. In addition, the ^1^H-NMR tracing of the H-D exchange reaction of methyl acetoacetate in MeOH-*d*_4_ illustrated that Hf(OTf)_4_ not only accelerated the tautomerization of methylacetoacetate but also activated the β-ketone moiety, thereby contributing to the overall promotion of Biginelli reaction. 

## Figures and Tables

**Table 1:**
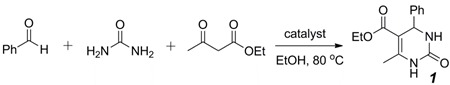
Catalytic effect of Group IVB transition metal Lewis acids on Biginelli reaction.

	Catalyst	Reaction Time (h)	Yield of 1 (%)
1	no	24	no reaction
2	conc. HCl	18	65
3	ZrCl_4_ (10 mol%)	12	80
4	ZrCp_2_Cl_2_ (10 mol%)	12	62 ^1^
5	ZrOCl_2_·8H_2_O (10 mol%)	9	78
6	HfCp_2_Cl_2_ (10 mol%)	12	73 ^1^
7	HfCl_4_ (10 mol%)	9	81
8	Hf(OTf)_4_ (10 mol%)	9	88

^1^ The starting materials were not completely consumed when the reaction reached equilibrium.

**Table 2:**
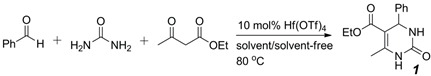
Solvent effect on the Hf(OTf)_4_-catalyzed Biginelli reaction.

	Solvent	Reaction Time (h)	Yield of 1 (%)
1	toluene	12	52 ^1^
2	1,2-dichloroethane	9	56 ^1^
3	THF	9	63 ^1^
4	CH_3_CN	9	65 ^1^
5	EtOH	9	88
6	solvent-free	5 min	96

^1^ The starting materials were not completely consumed when the reaction reached equilibrium.

**Table 3:**
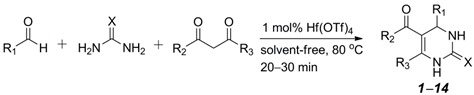
Hf(OTf)_4_-catalyzed synthesis of substituted DHPMs **1**–**14** under solvent-free conditions.

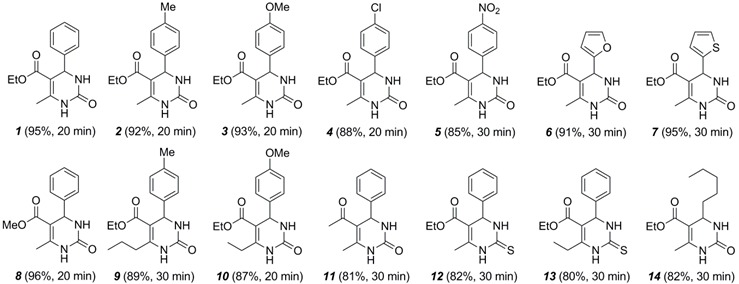

## Supplementary Materials

The following are available online, Figures S1–S42: The NMR spectra of compounds **1**–**17** and **19**–**21**; Figures S43–S44: The ^1^H-NMR tracing of the H-D exchange reactions of methyl acetoacetate in MeOH-*d*_4_.
