# Supplementary material for: Highly Efficient Synthesis of Substituted 3,4-Dihydropyrimidin-2-(1H)-ones (DHPMs) Catalyzed by Hf(OTf)4: Mechanistic Insights into Reaction Pathways under Metal Lewis Acid Catalysis and Solvent-Free Conditions
Source: Molecules. 2019 Jan 21;24(2):364. doi: 10.3390/molecules24020364 (PMC6359175; doi:10.3390/molecules24020364)
Supplement: Supplementary file 1 [file molecules-24-00364-s001.pdf]

Supporting Information

Highly efficient synthesis of substituted 3,4-dihydropyrimidin-2-(1*H*)-ones (DHPMs) catalyzed by $Hf(OTf)_4$: Mechanistic insights into reaction pathways under metal Lewis acid catalysis and solvent-free conditions

Rui Kong, Shuai-Bo Han, Jing-Ying Wei, Xiao-Chong Peng, Zhen-Biao Xie,
Shan-Shan Gong* and Qi Sun*

Jiangxi Key Laboratory of Organic Chemistry, Jiangxi Science and Technology Normal University, 605 Fenglin Avenue, Nanchang, Jiangxi 330013, PR China

E-mails: gongshanshan@jxstnu.edu.cn;sunqi@jxstnu.edu.cn

Table of contents

1. The NMR spectra of DHPMs **1**–**14** and **20**	Page S2–S16
2. The NMR spectra of compounds **15**–**17**, **19** and **21**	Page S17–S22
3. The ^{1}H-NMR tracing of H-D exchange reactions of methyl acetoacetate in MeOH-d_4	Page S23

1. The NMR spectra of DHPMs 1–14 and 20

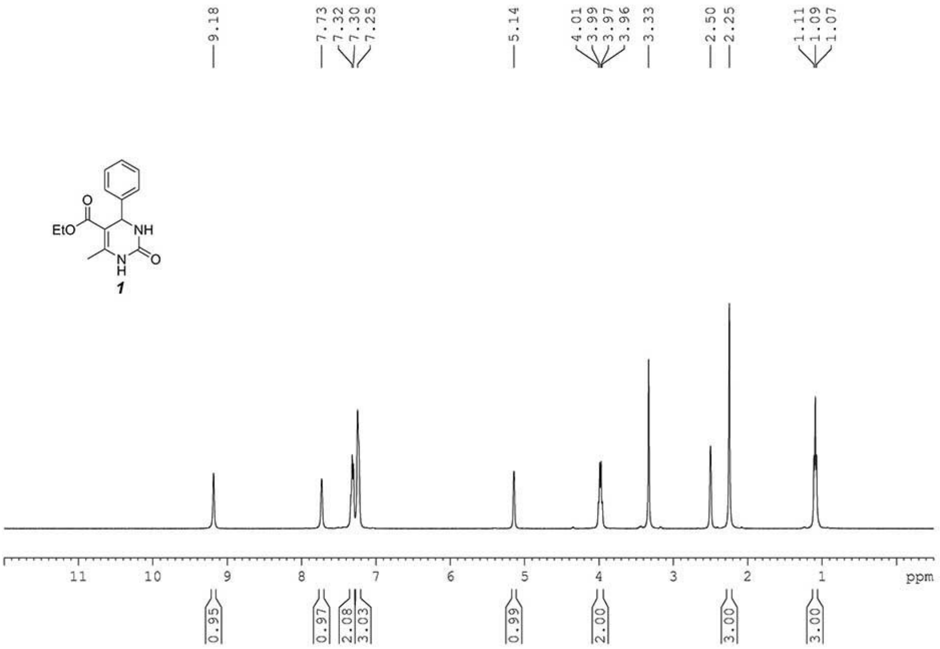

Figure S1. ^{1}H-NMR of **1**

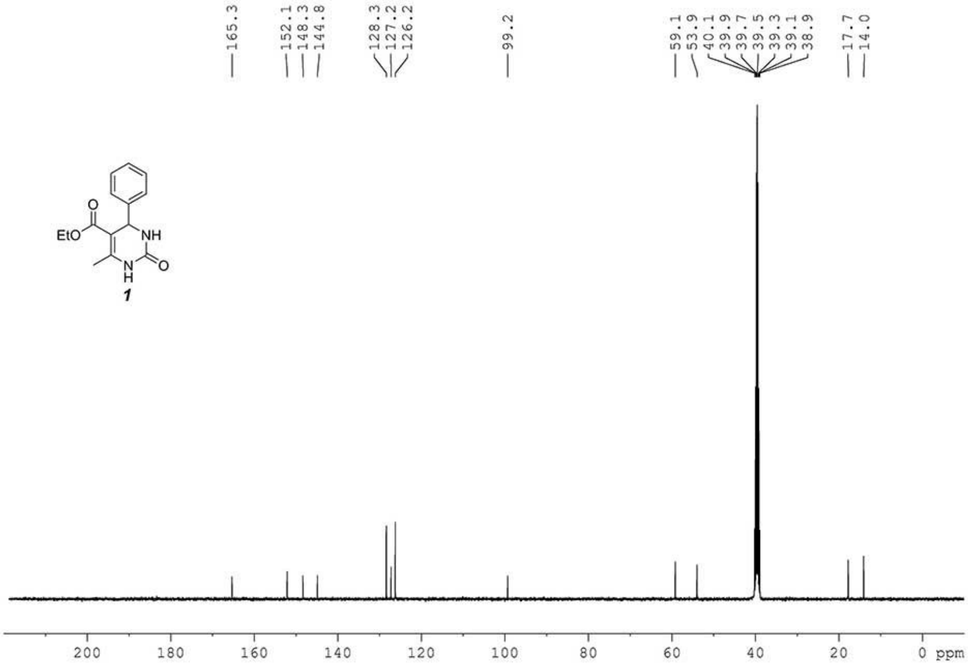

Figure S2. ^{13}C-NMR of **1**

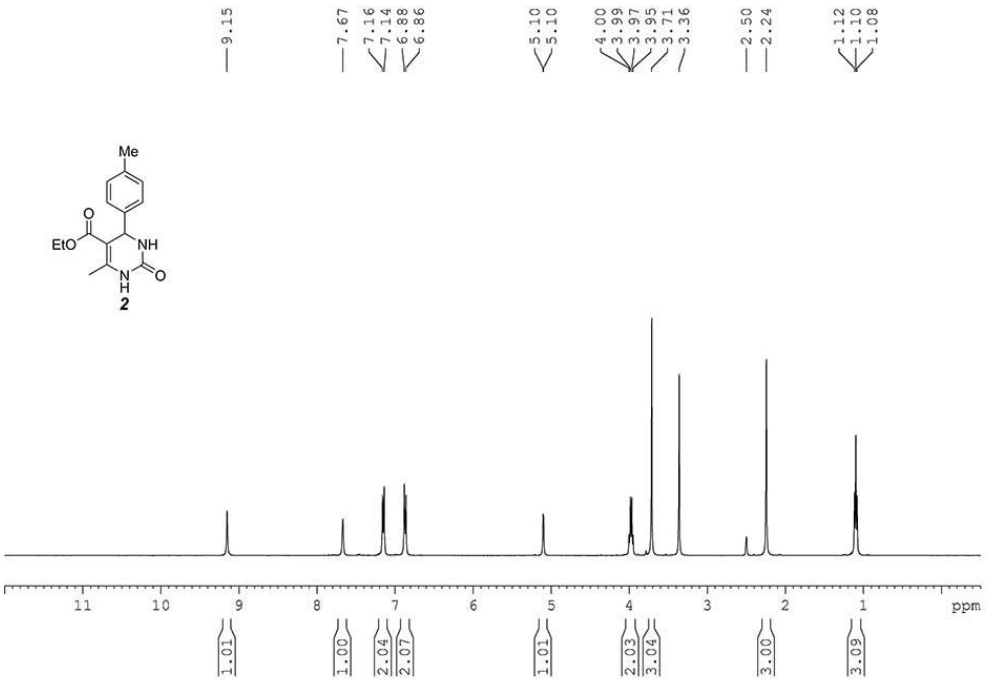

Figure S3. ^{1}H-NMR of **2**

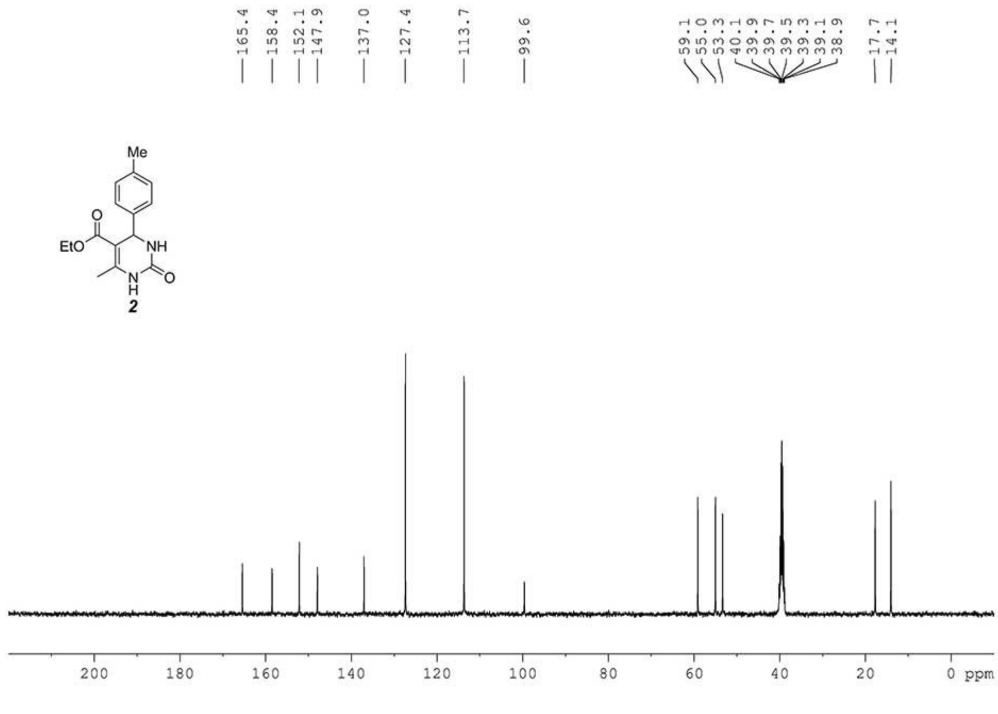

Figure S4. ^{13}C-NMR of **2**

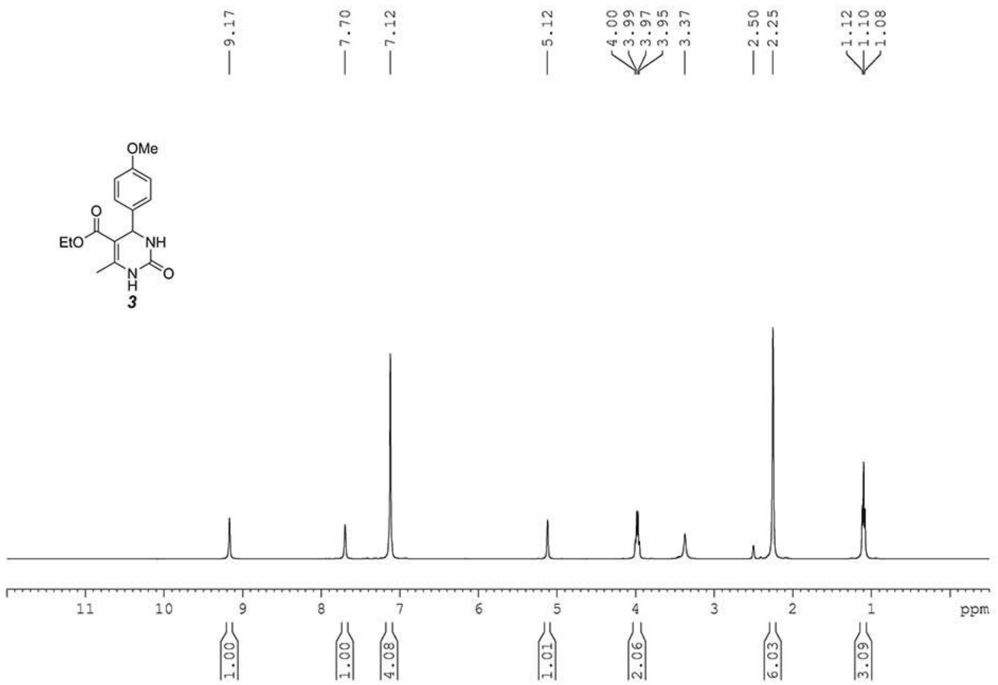

Figure S5. ^{1}H-NMR of **3**

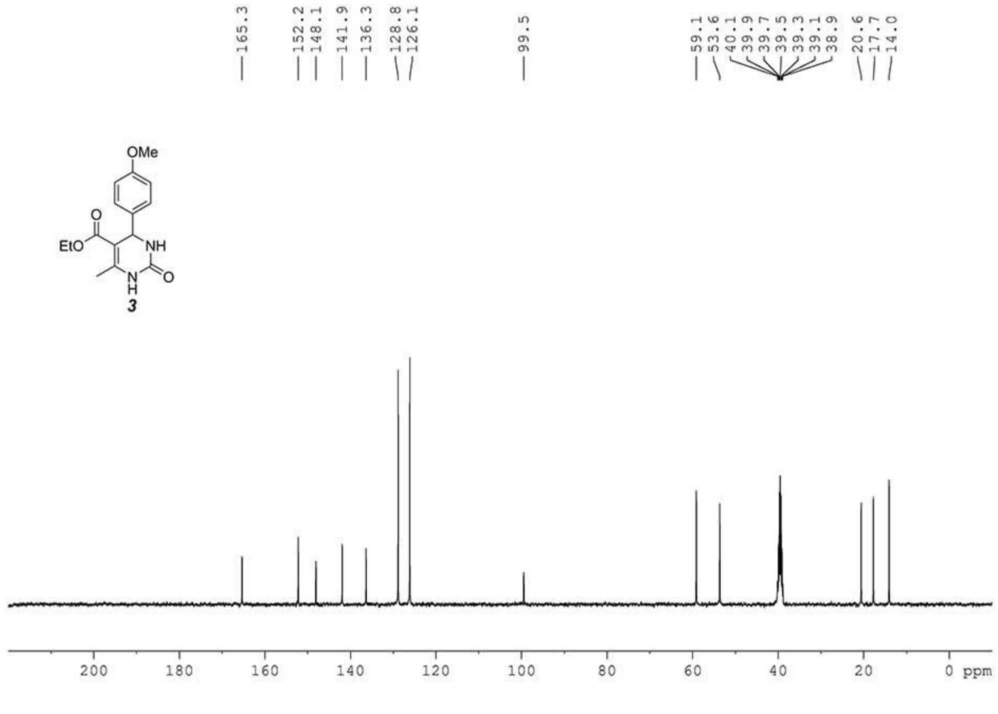

Figure S6. ^{13}C-NMR of **3**

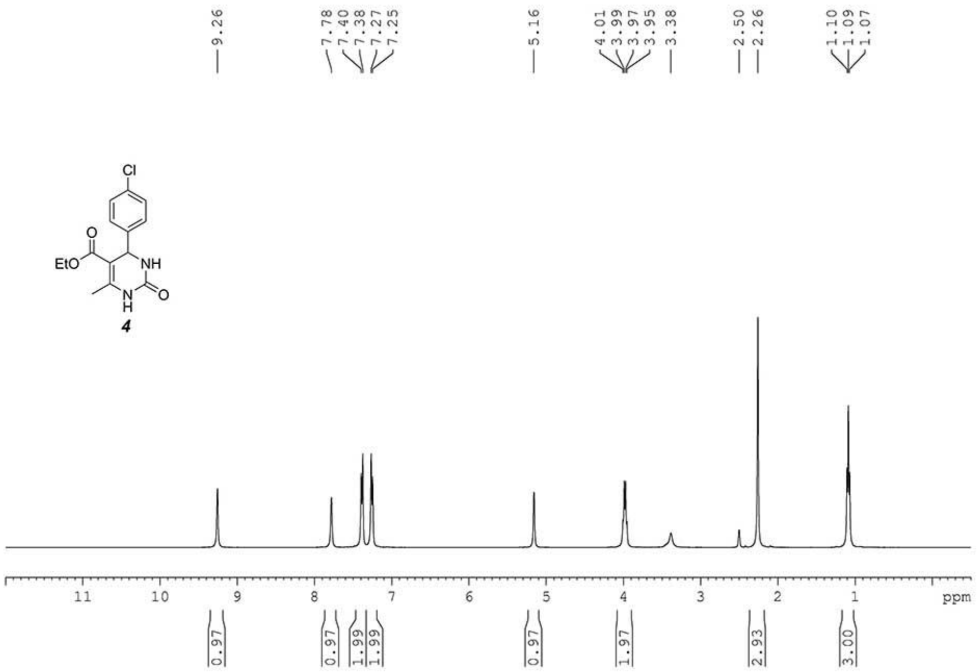

Figure S7. ^{1}H-NMR of **4**

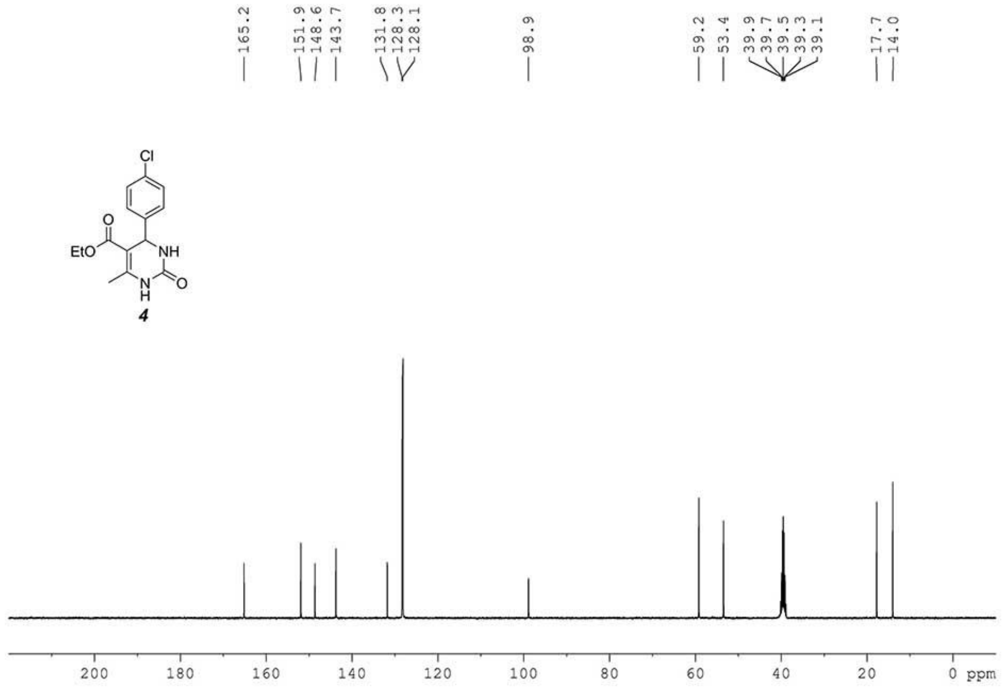

Figure S8. ^{13}C-NMR of **4**

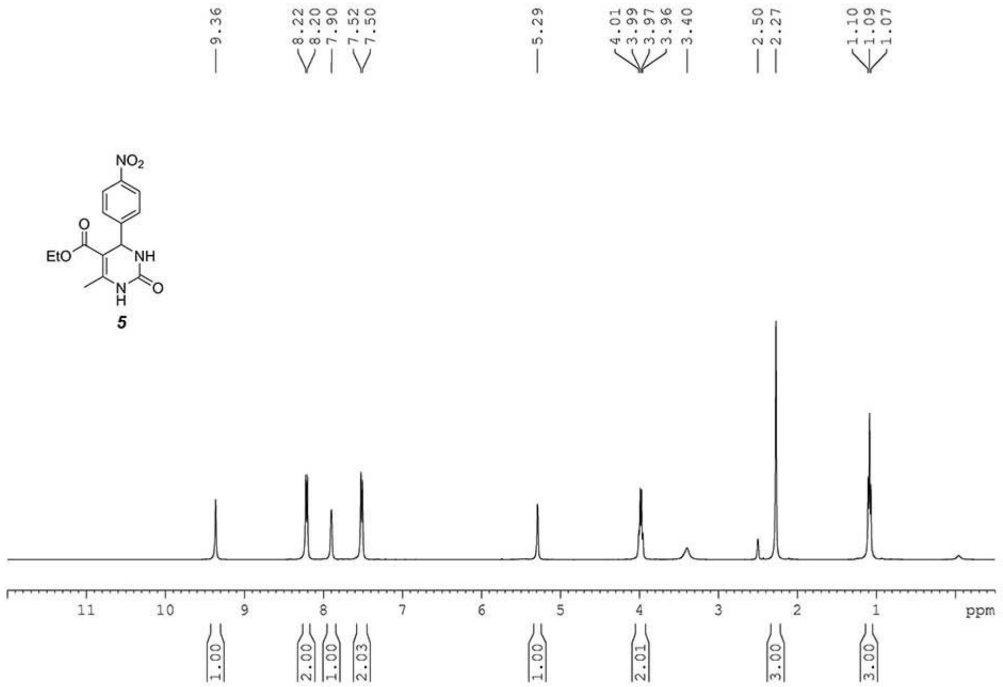

Figure S9. ^{1}H-NMR of **5**

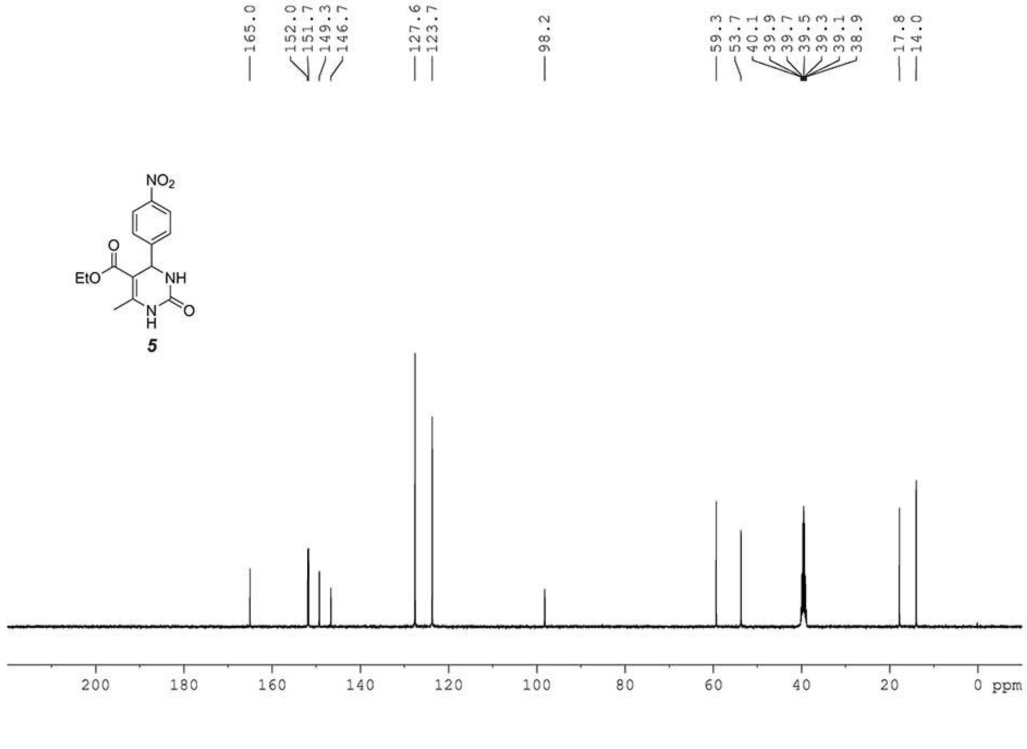

Figure S10. ^{13}C-NMR of **5**

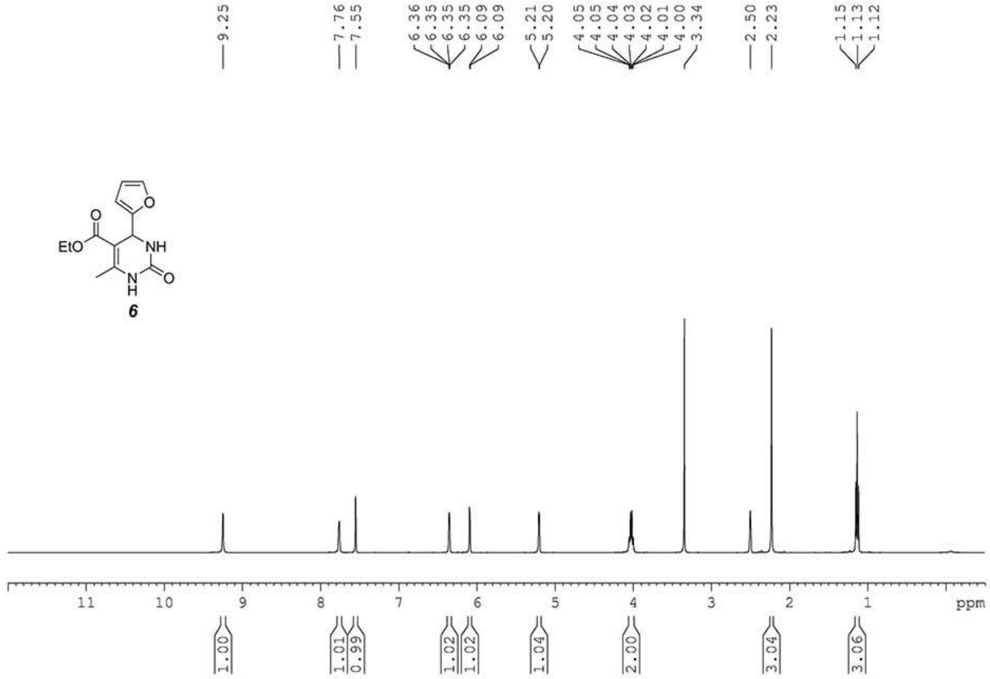

Figure S11. ^{1}H-NMR of **6**

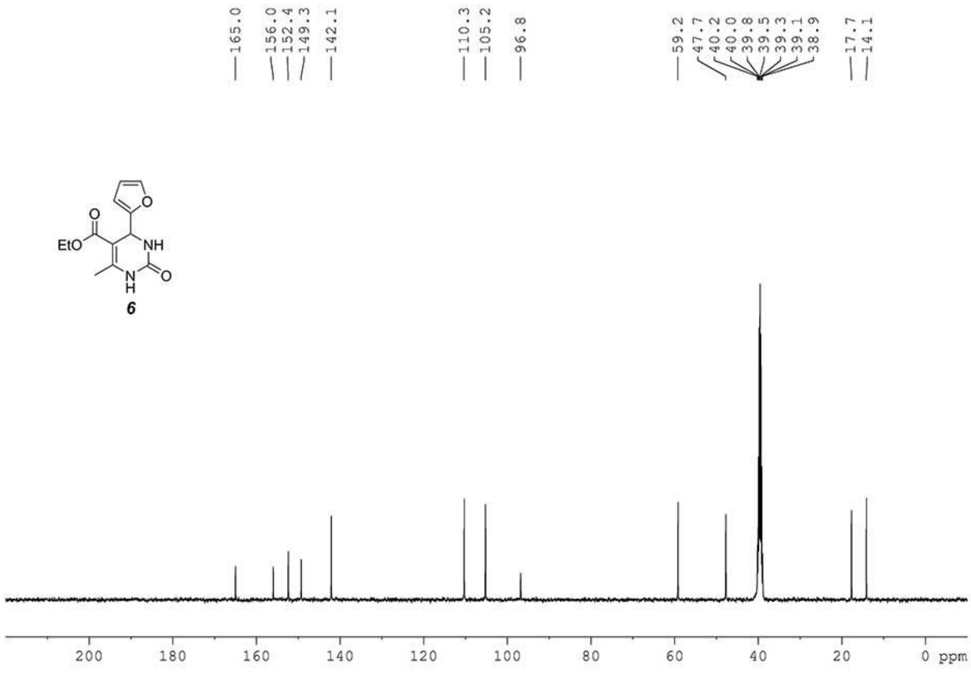

Figure S12. ^{13}C-NMR of **6**

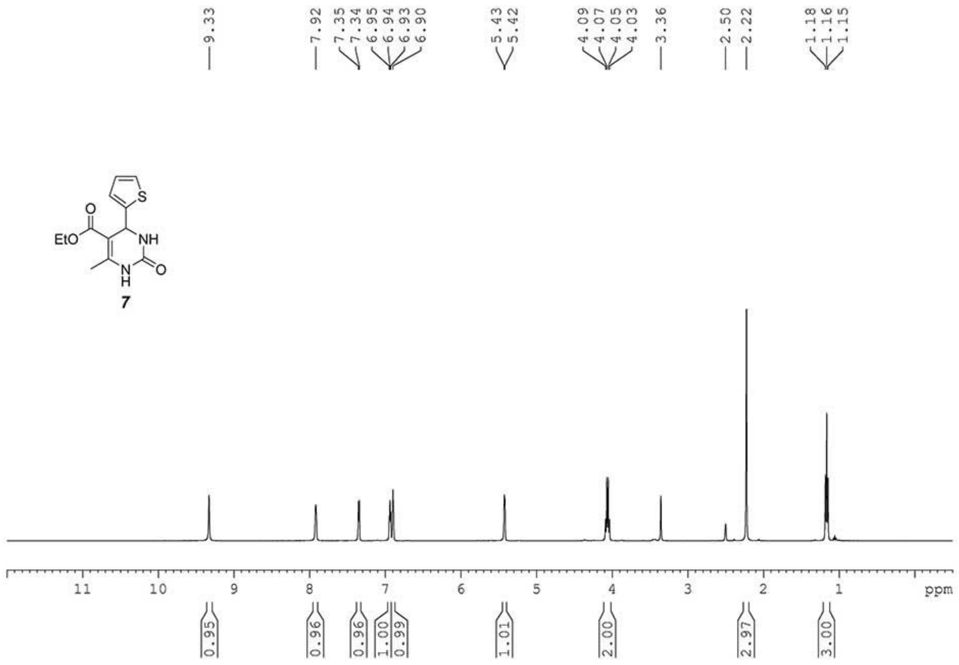

Figure S13. ^{1}H-NMR of **7**

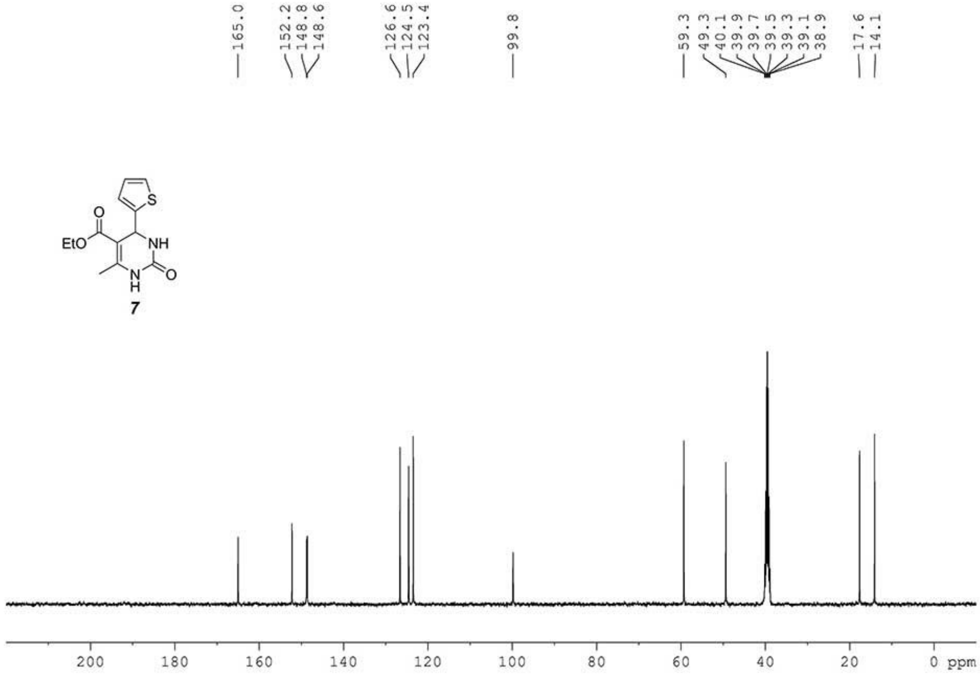

Figure S14. ^{13}C-NMR of **7**

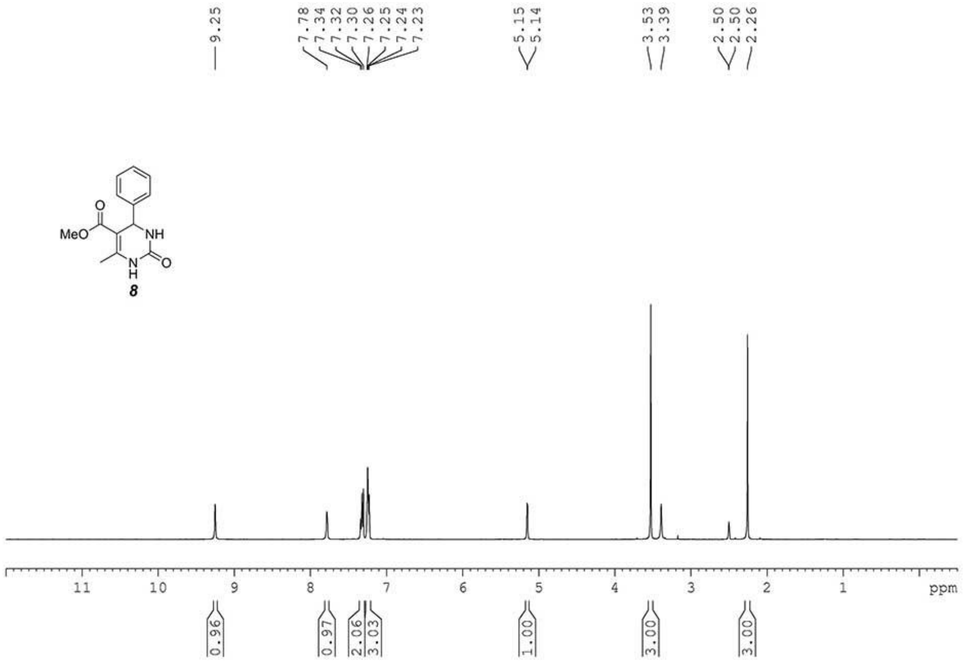

Figure S15. ^{1}H-NMR of **8**

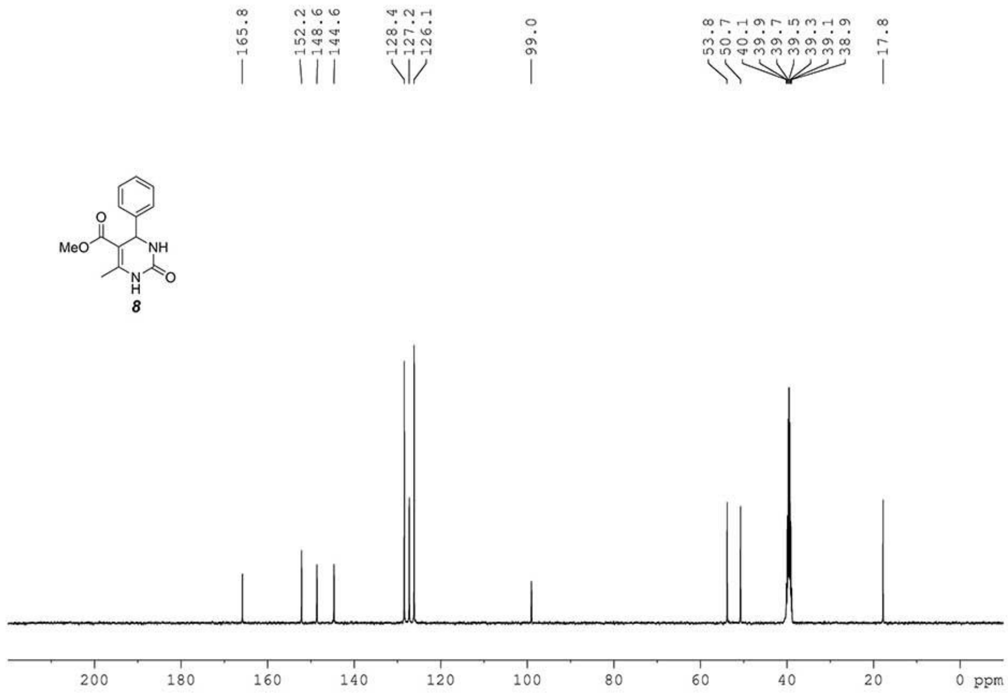

Figure S16. ^{13}C-NMR of **8**

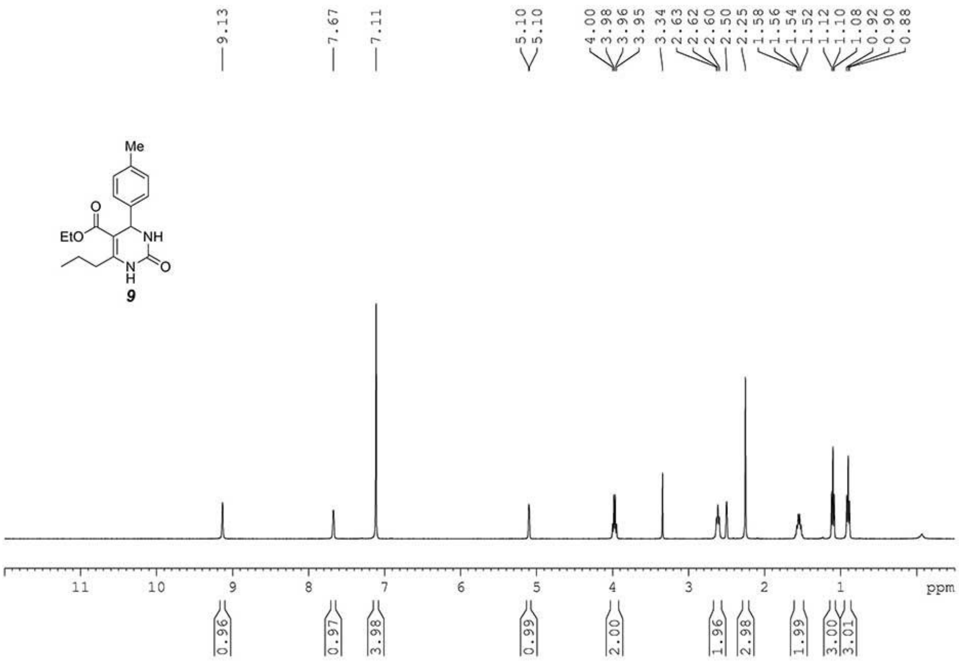

Figure S17. ^{1}H-NMR of **9**

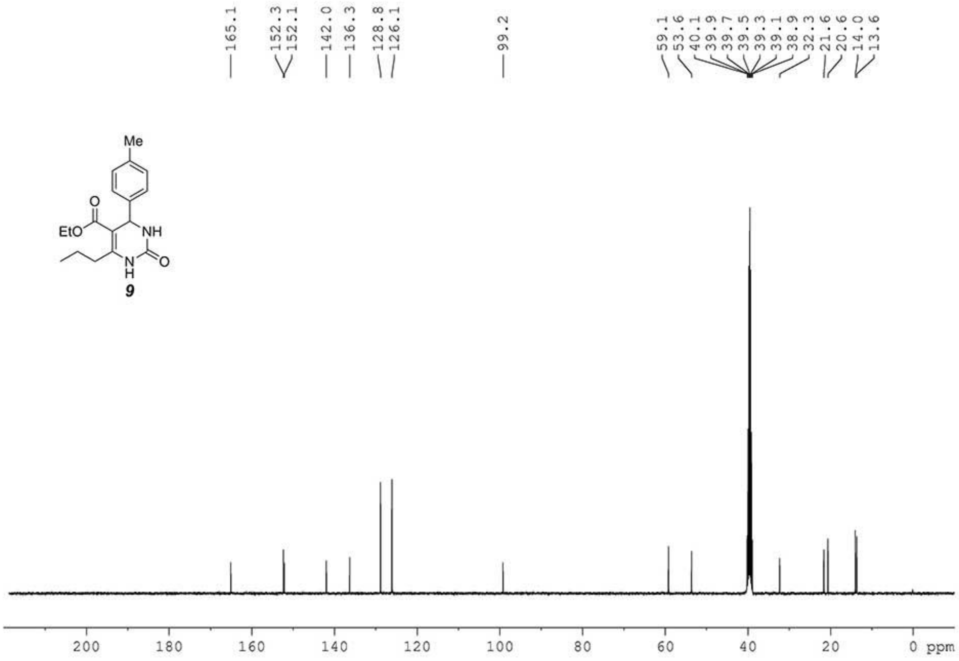

Figure S18. ^{13}C-NMR of **9**

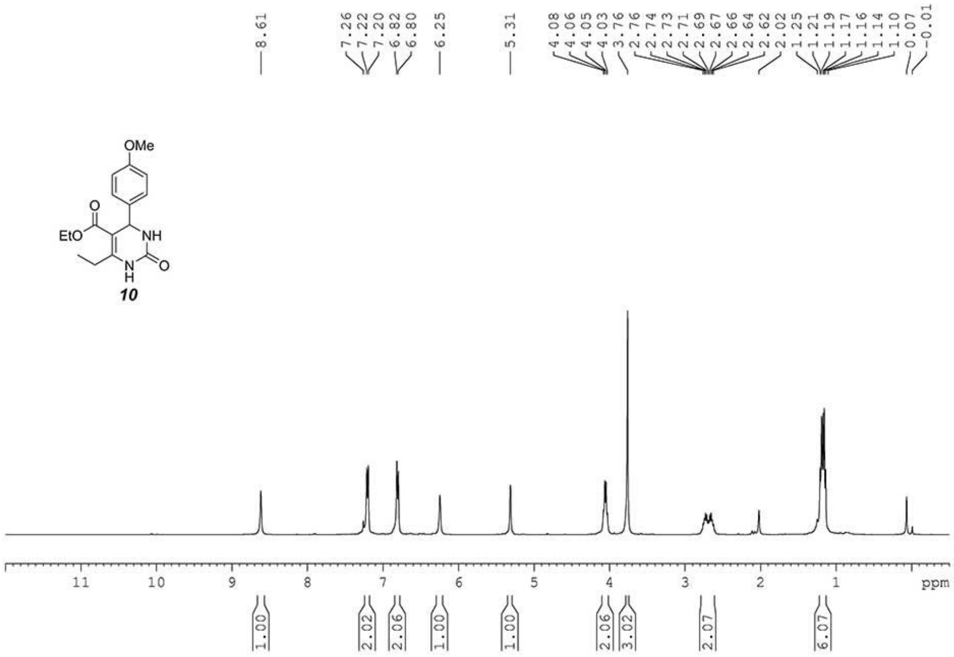

Figure S19. ^{1}H-NMR of **10**

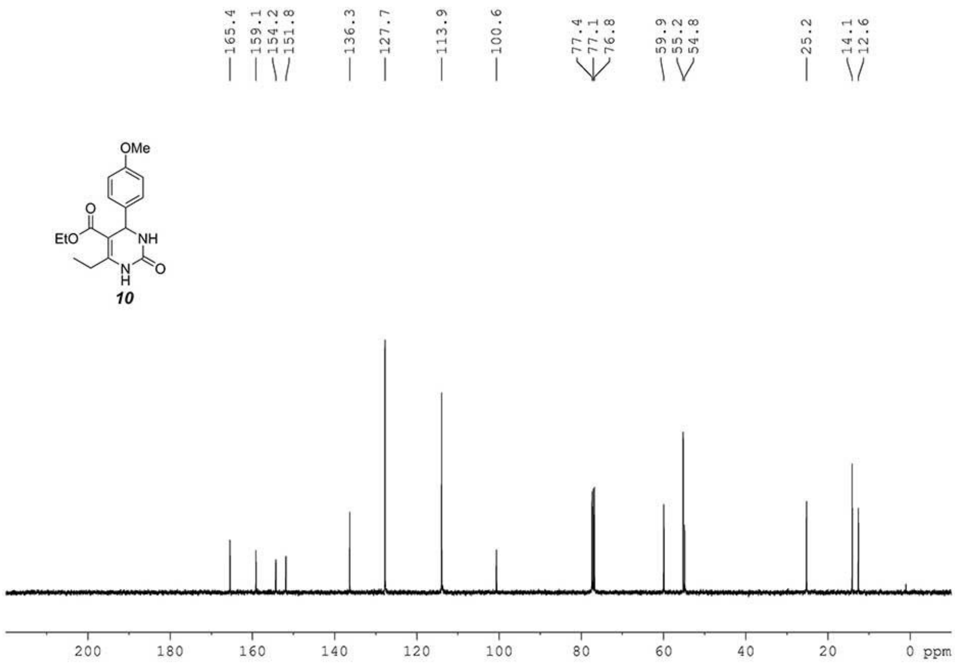

Figure S20. ^{13}C-NMR of **10**

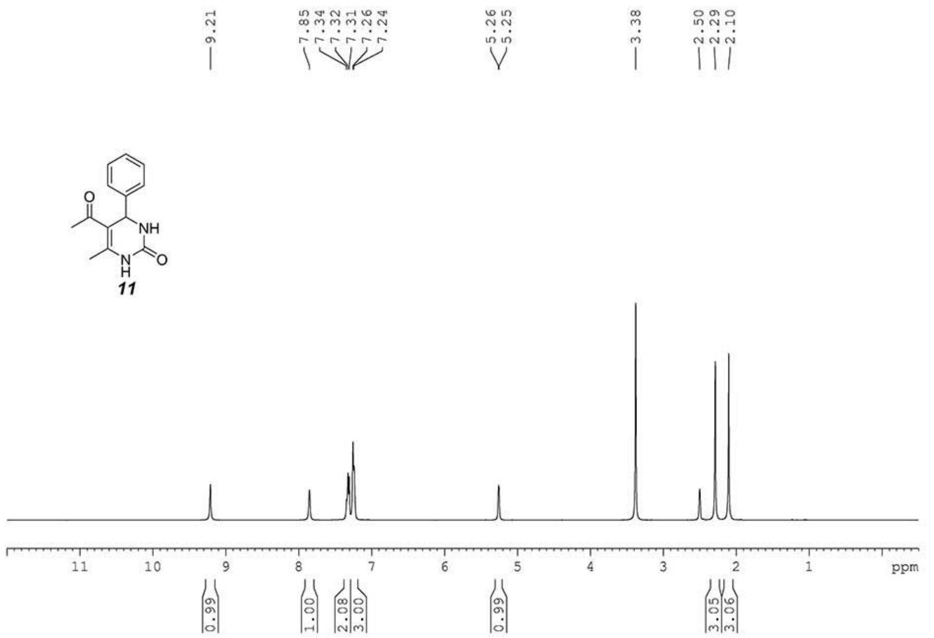

Figure S21. ^{1}H-NMR of **11**

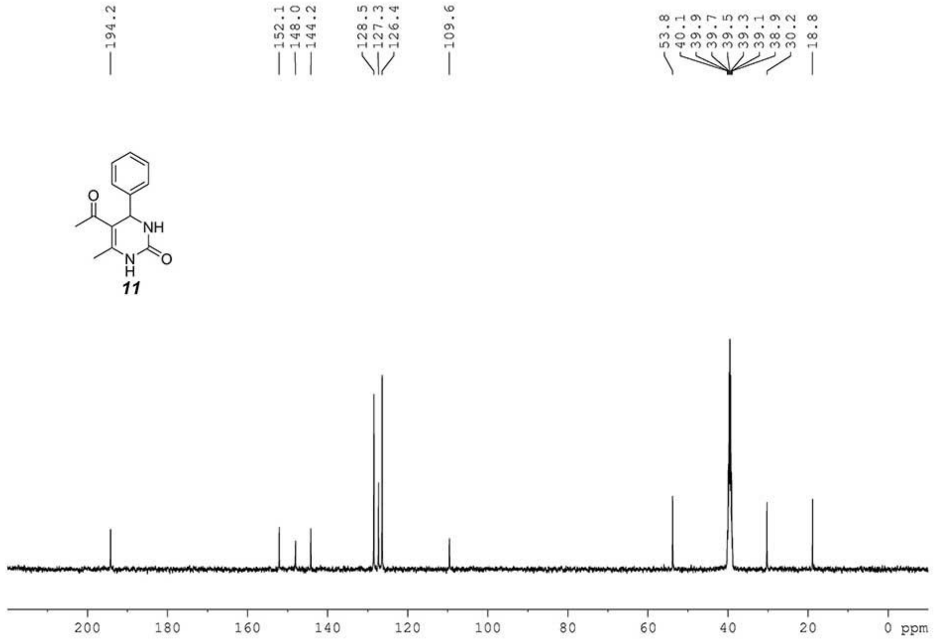

Figure S22. ^{13}C-NMR of **11**

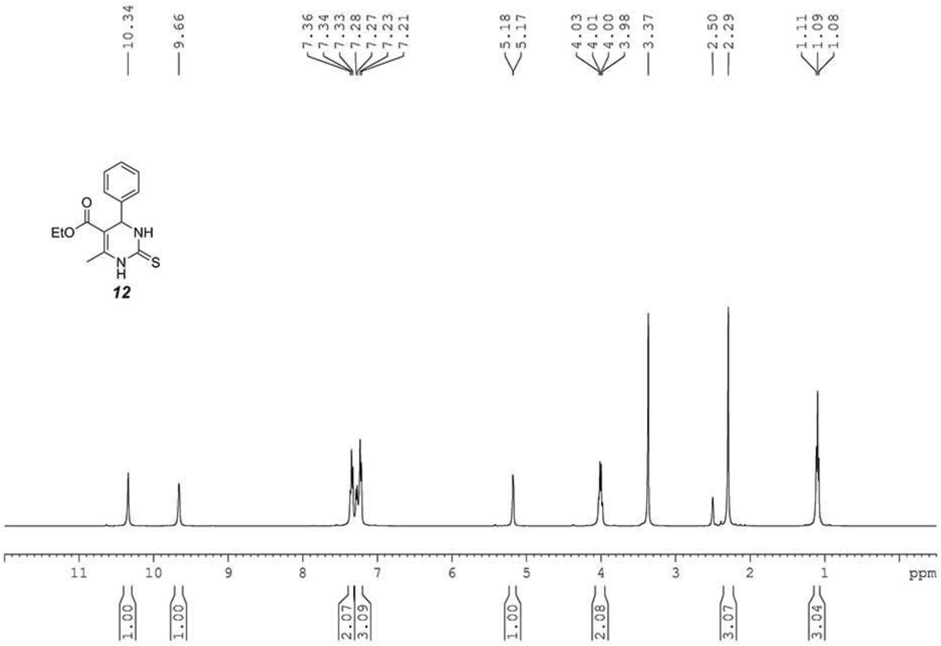

Figure S23. ^{1}H-NMR of **12**

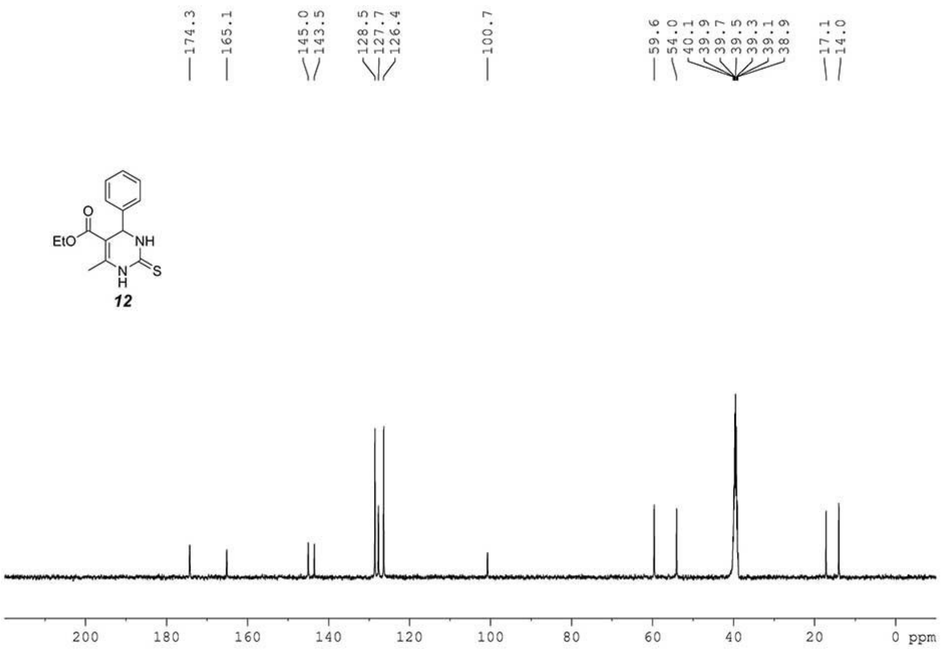

Figure S24. ^{13}C-NMR of **12**

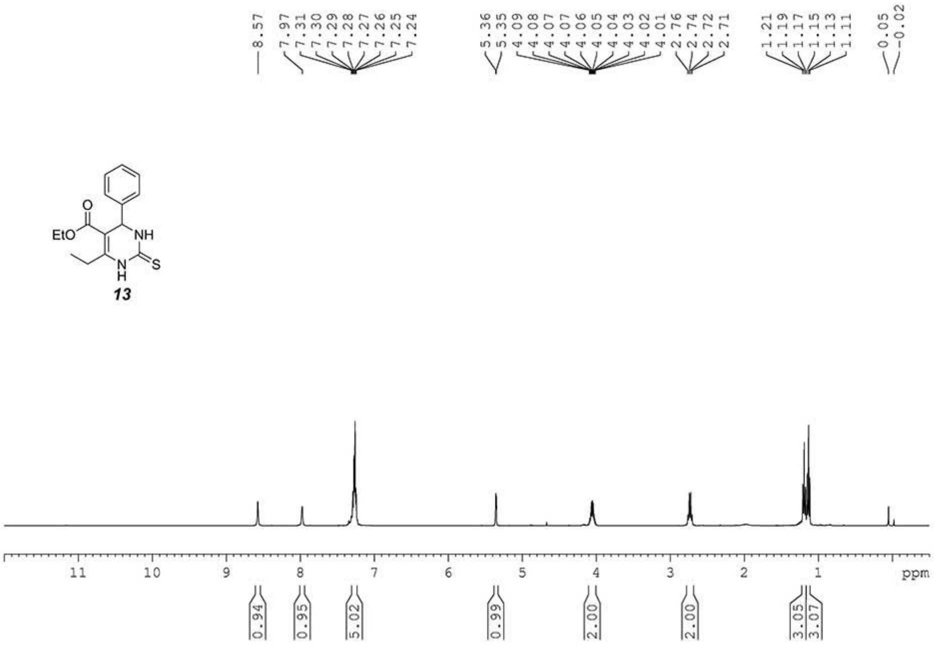

Figure S25. ^{1}H-NMR of **13**

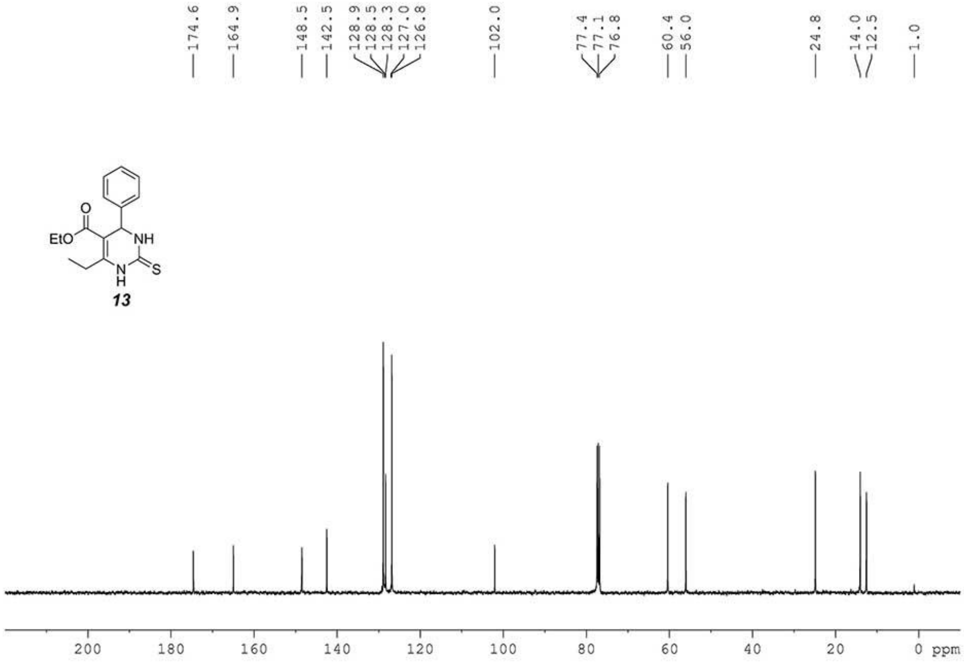

Figure S26. ^{13}C-NMR of **13**

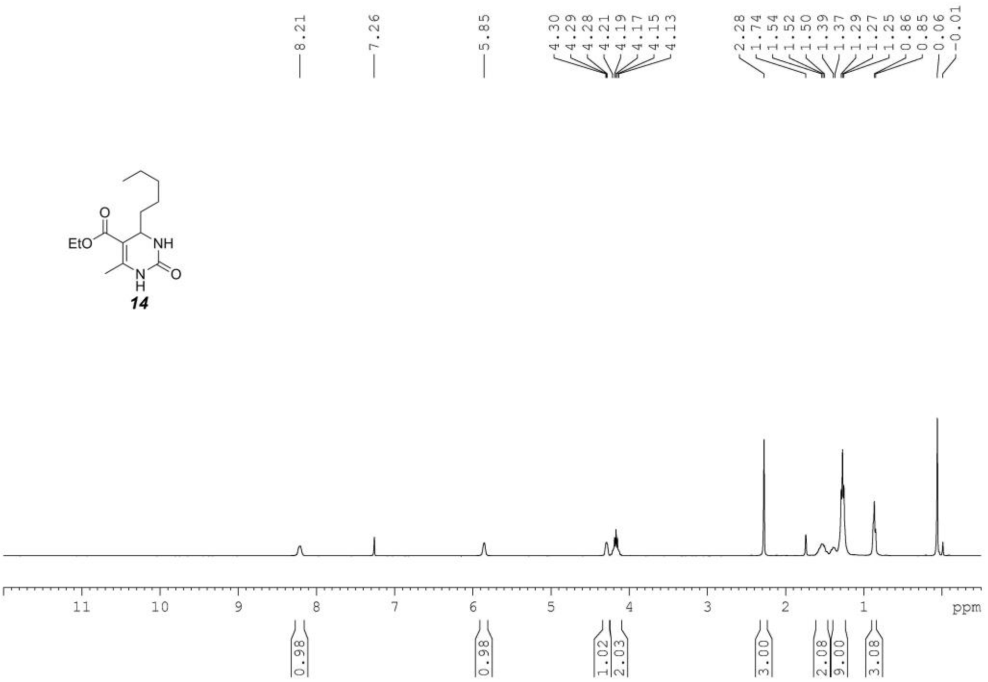

Figure S27. ^{1}H-NMR of **14**

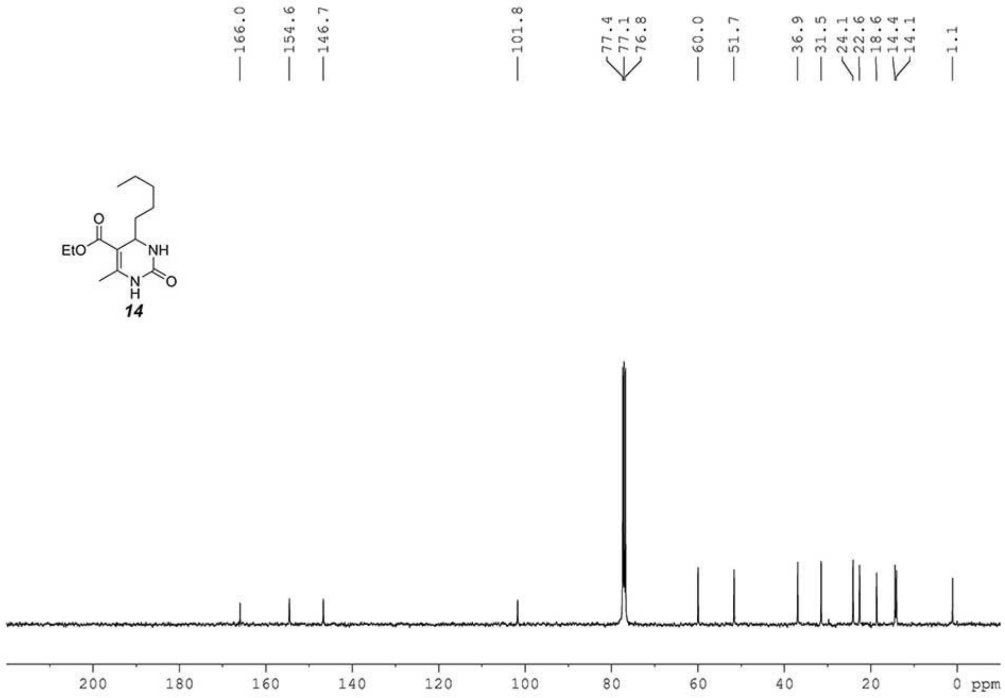

Figure S28. ^{13}C-NMR of **14**

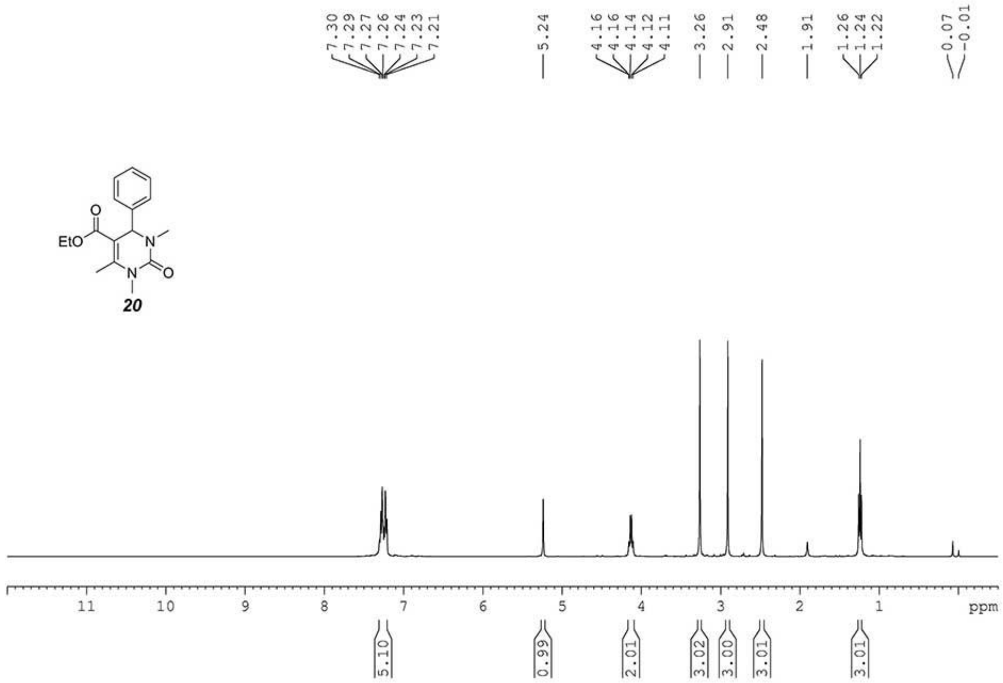

Figure S29. ^{1}H-NMR of **20**

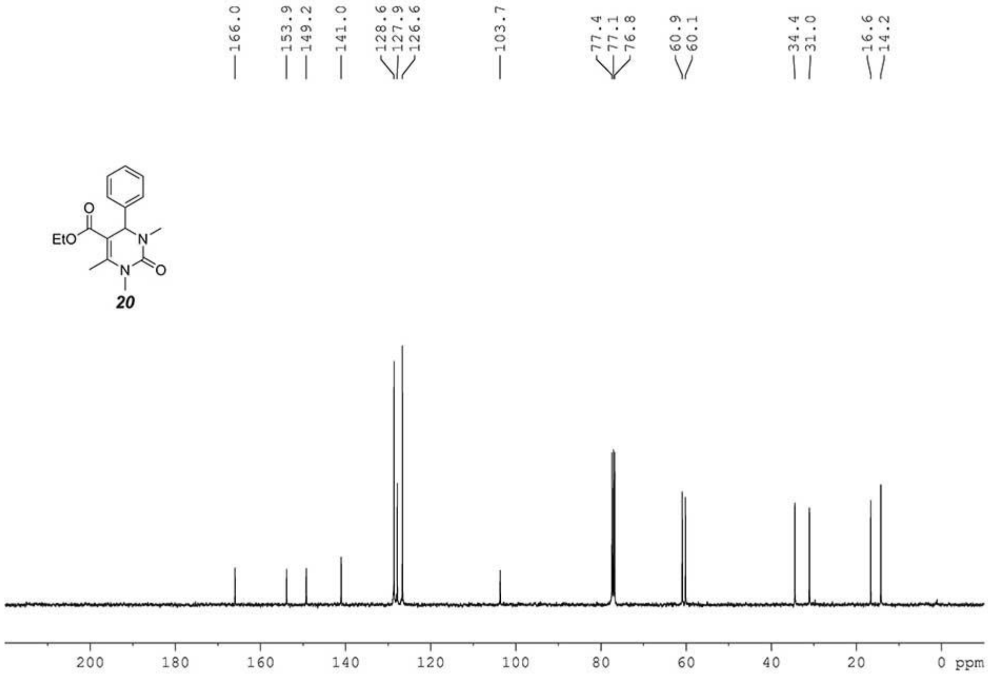

Figure S30. ^{13}C-NMR of **20**

2. The NMR spectra of compounds (15-17, 19, and 21).

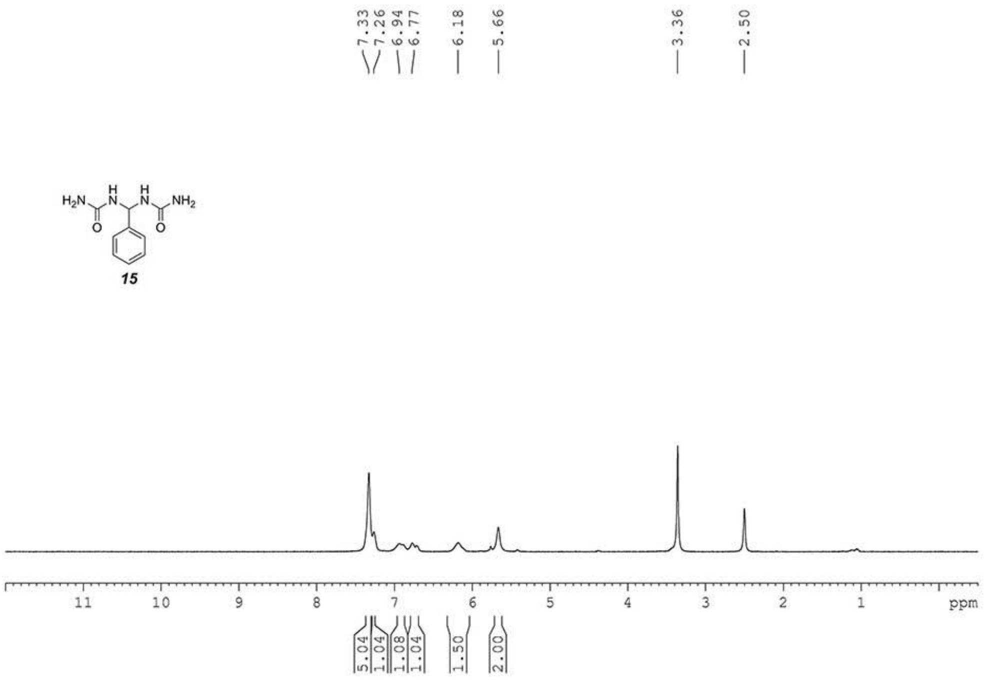

Figure S31. ^{1}H-NMR of **15**

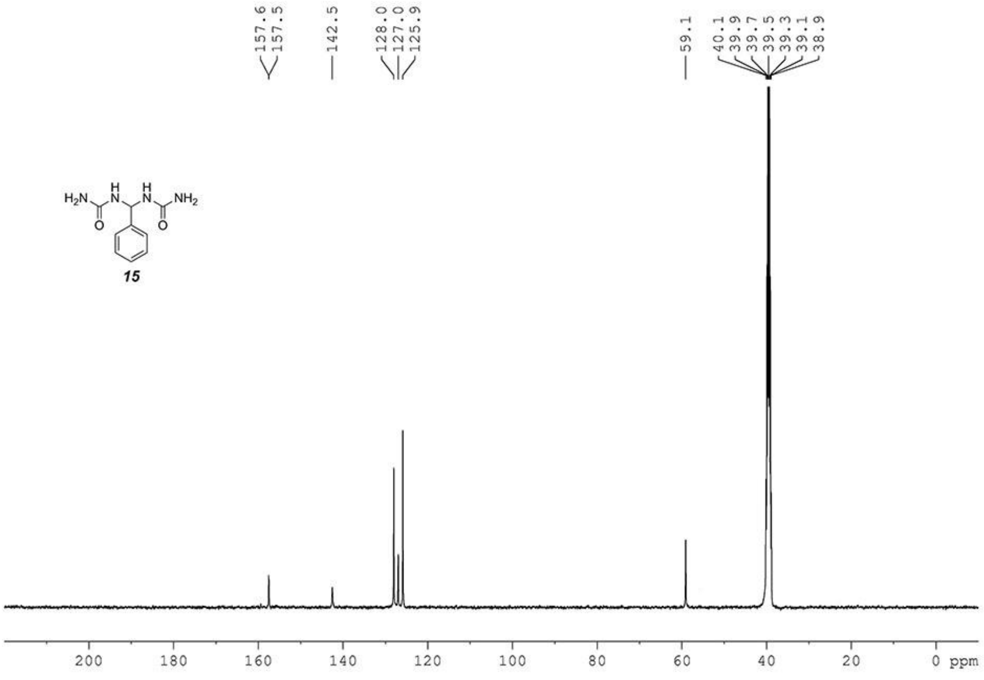

Figure S32. ^{13}C-NMR of **15**

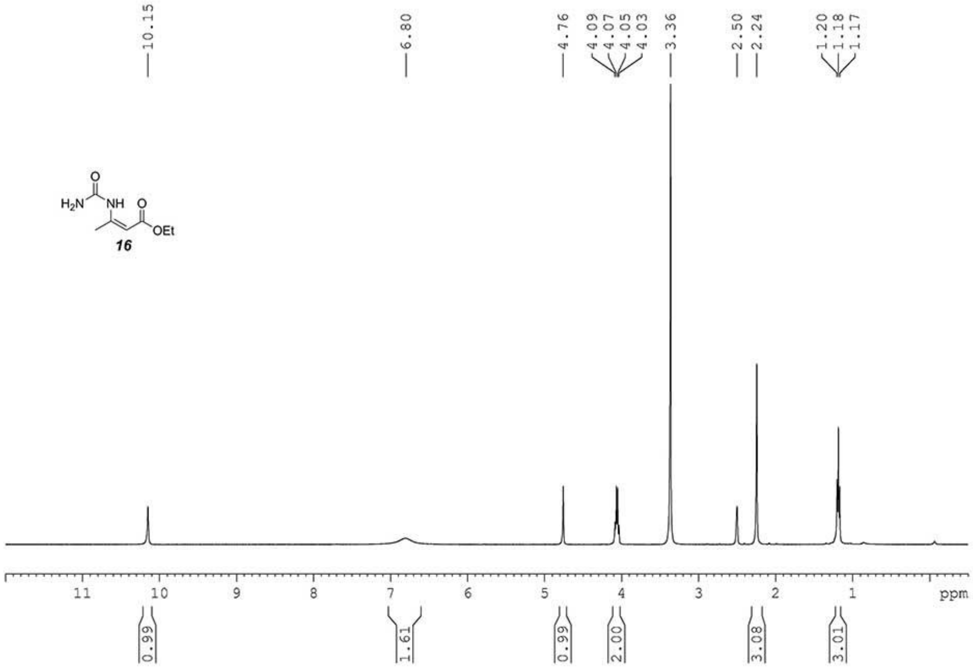

Figure S33. ^{1}H-NMR of **16**

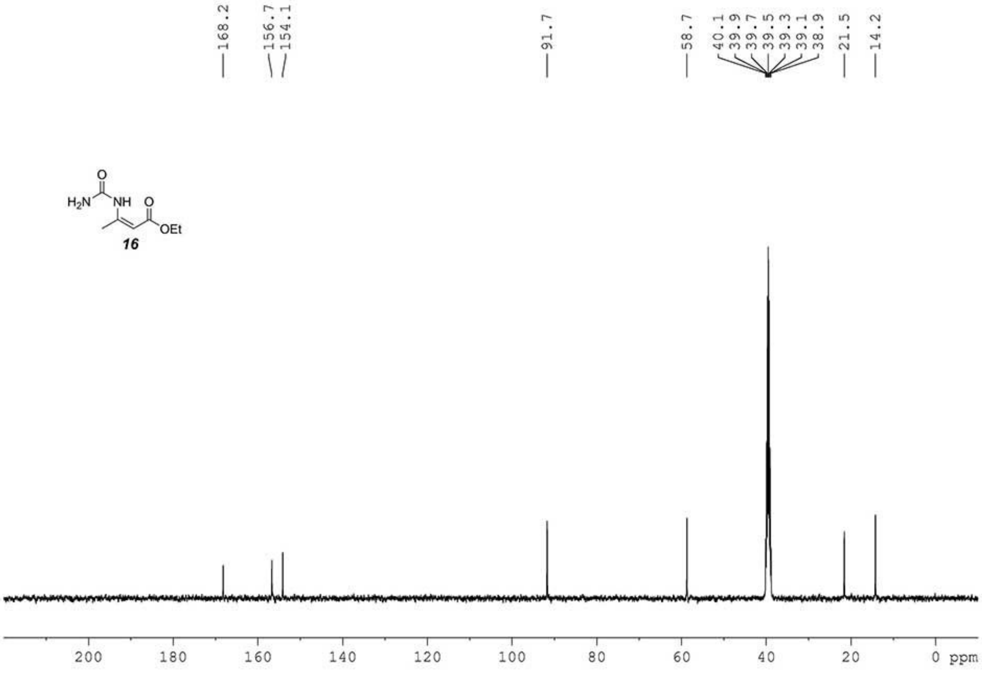

Figure S34. ^{13}C-NMR of **16**

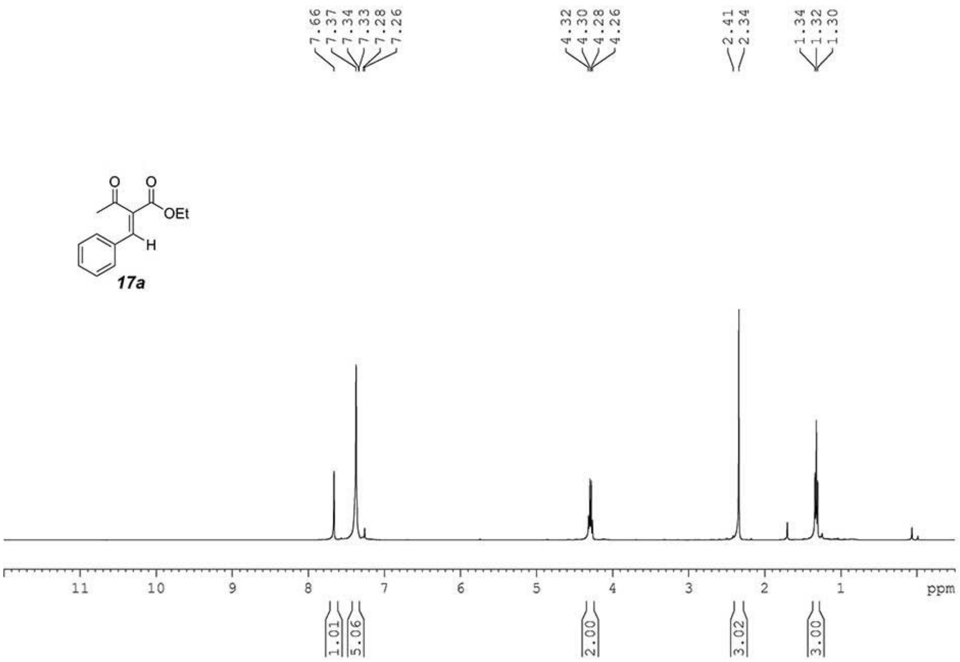

Figure S35. ^{1}H-NMR of **17a**

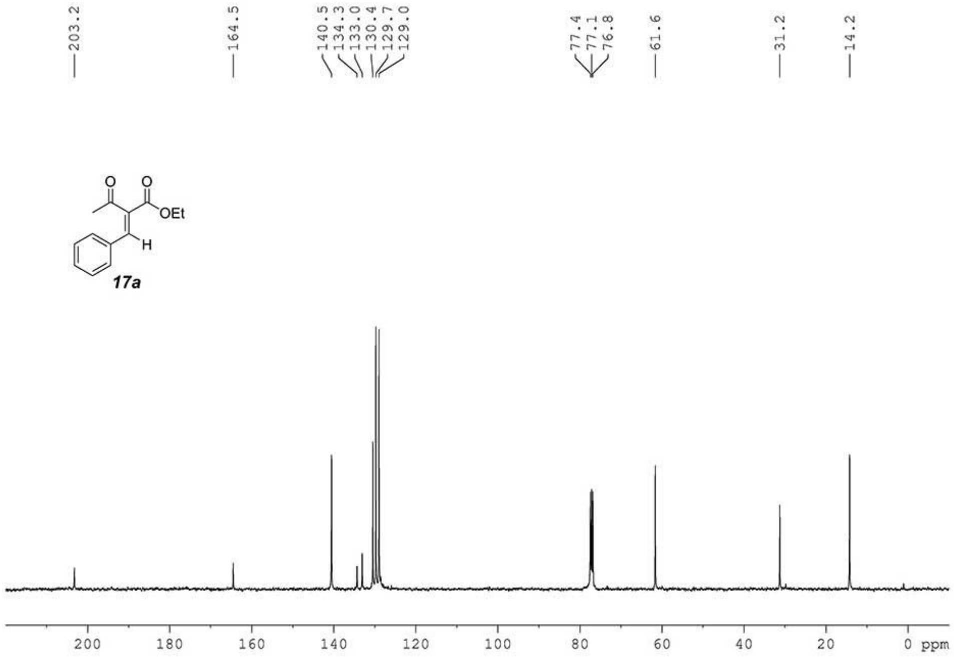

Figure S36. ^{13}C-NMR of **17a**

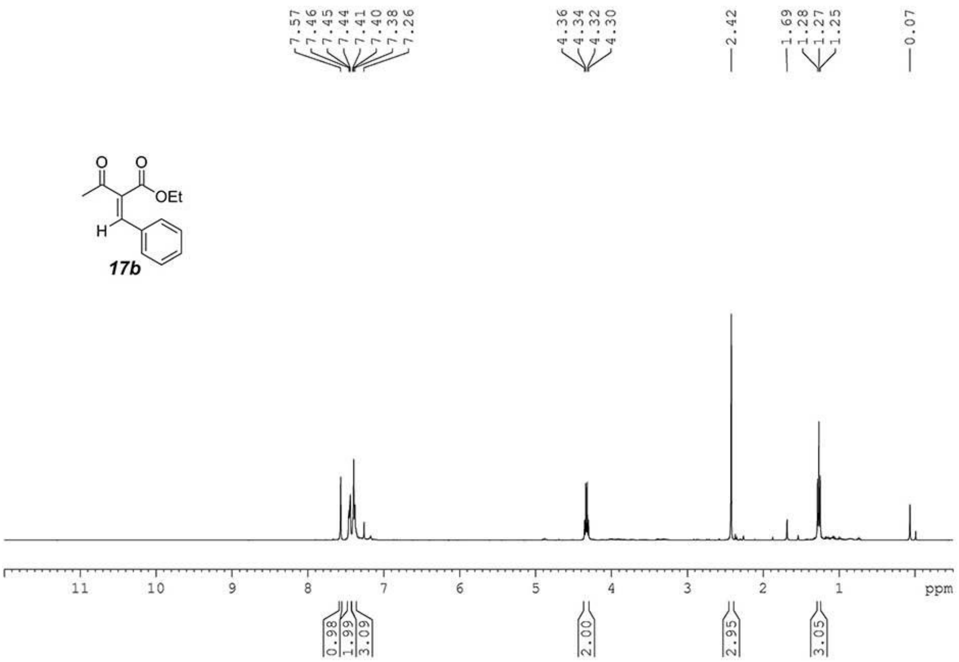

Figure S37. ^{1}H-NMR of **17b**

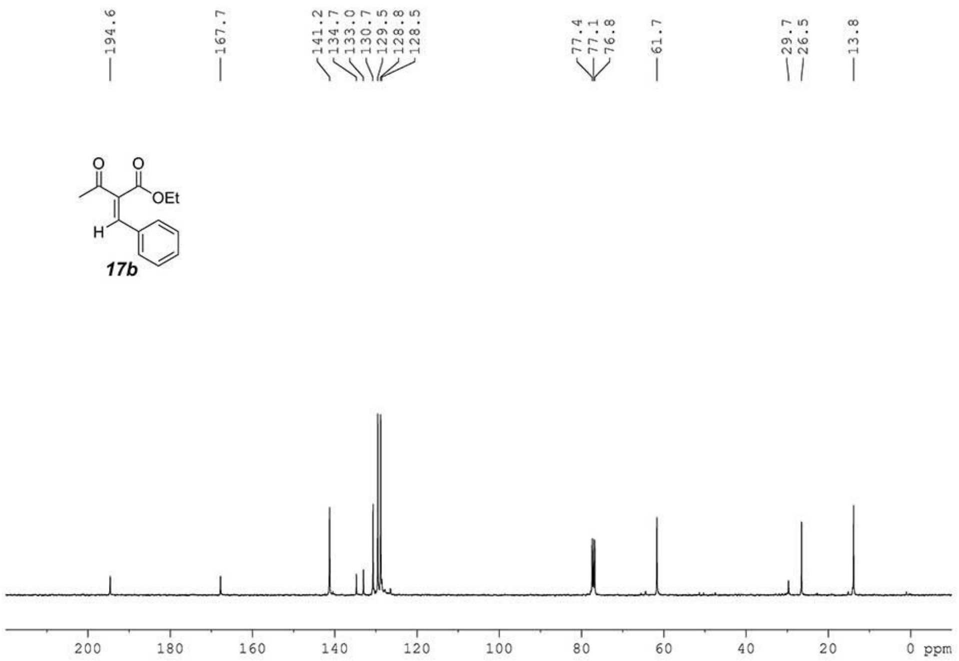

Figure S38. ^{13}C-NMR of **17b**

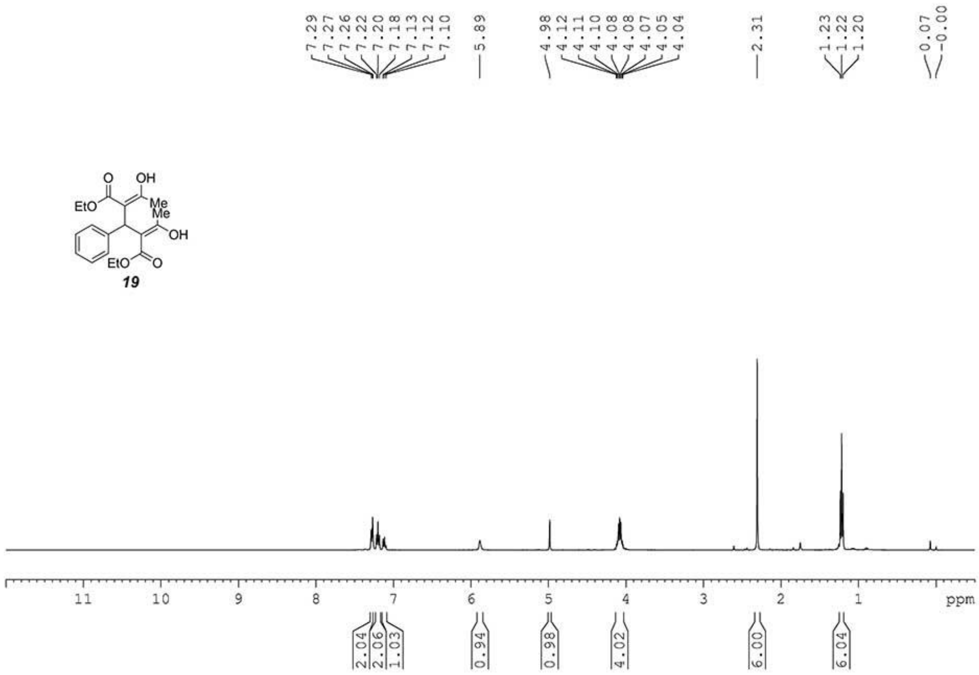

Figure S39. ^{1}H-NMR of **19**

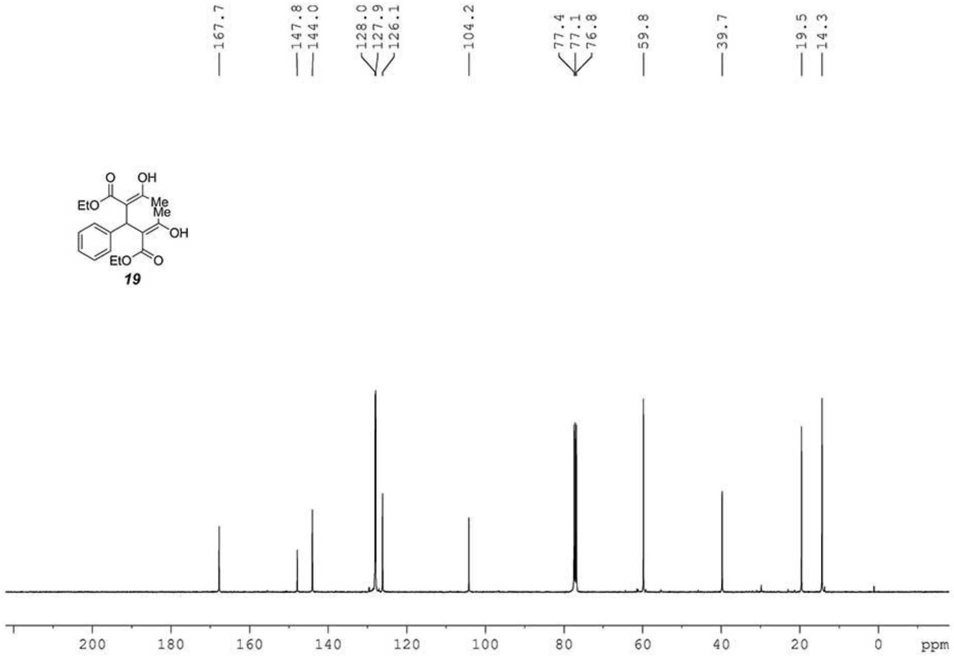

Figure S40. ^{13}C-NMR of **19**

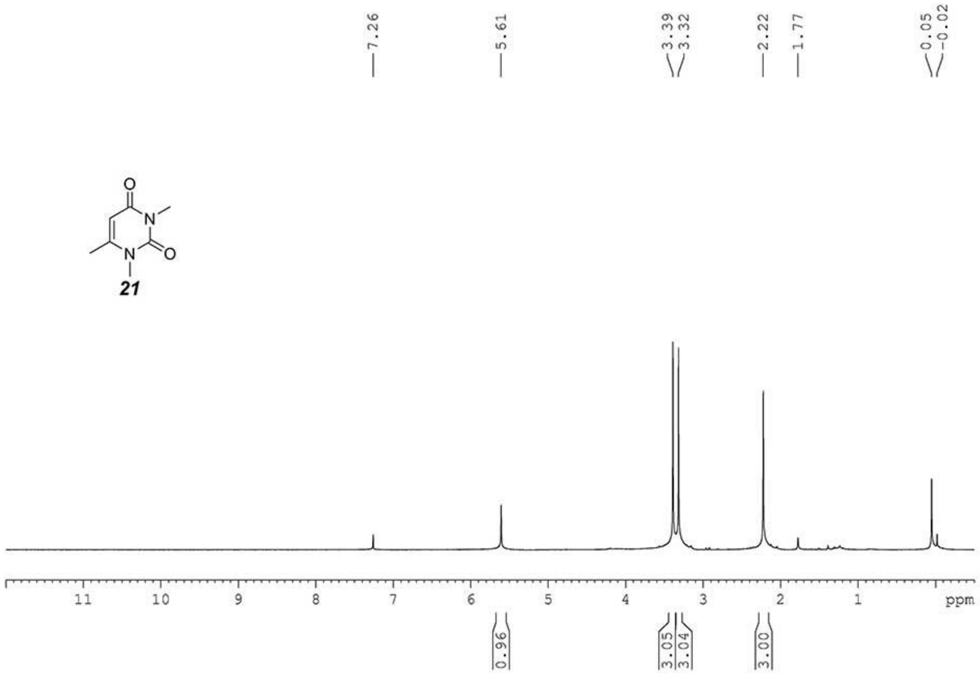

Figure S41. ^{1}H-NMR of **21**

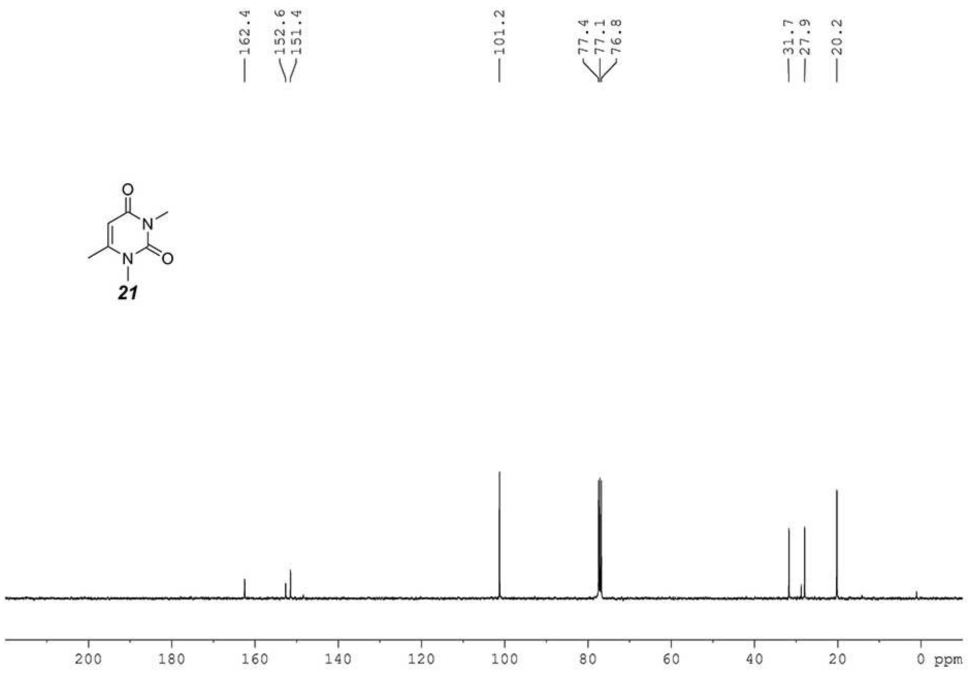

Figure S42. ^{13}C-NMR of **21**

3. The ^{1}H-NMR tracing of the H-D exchange reactions of methyl acetoacetate in MeOH-d_4

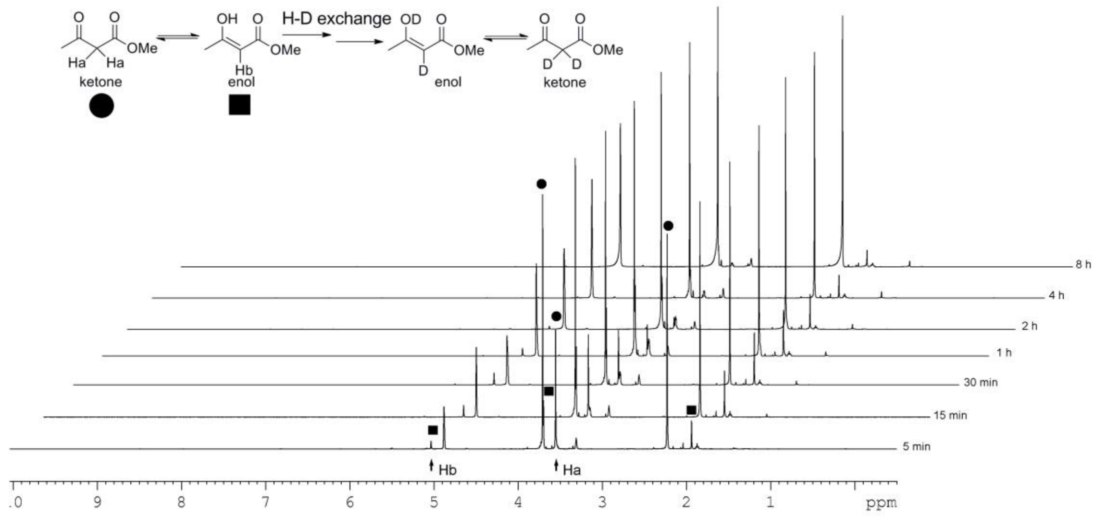

Figure S43. ^{1}H-NMR of H-D exchange reaction of methyl acetoacetate in MeOH-d_4 without $Hf(OTf)_4$

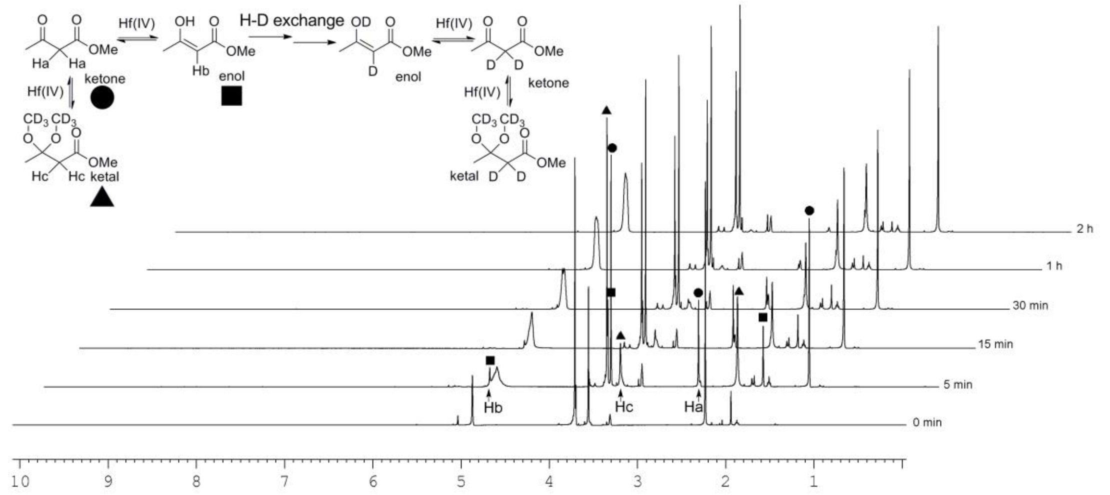

Figure S44. ^{1}H-NMR of H-D exchange reaction of methyl acetoacetate in MeOH-d_4 with 5 mol% $Hf(OTf)_4$
